# Research on the path of high-quality development of the smart health and aging care industry driven by digital economy— dynamic QCA analysis based on TOE framework

**DOI:** 10.3389/fpubh.2026.1661333

**Published:** 2026-03-02

**Authors:** Xinhua Tan, Xiaobing Shu

**Affiliations:** School of Public Administration, Central China Normal University, Wuhan, Hubei, China

**Keywords:** digital economy, smart health and aging care industry, high quality development, TOE framework, dynamic QCA, grouping paths

## Abstract

**Introduction:**

The digital economy has become a important driver for the high-quality development of industry. Exploring its impact on and pathways for enhancing the smart health and aging care industry holds significant practical importance.

**Methods:**

Leveraging the theoretical underpinnings of TOE framework and configuration analysis, this research utilizes dynamic Qualitative Comparative Analysis (QCA) to examine provincial-level panel data from China between 2012–2022, investigating how the digital economy’s configuration effects evolve over time.

**Results:**

The digital economy drives the high-quality development of the smart health and aging care industry through five pathways, which can be divided into three classes: digitally technology-driven, dual-core “digital technology–digital environment” driven, and digitally environment-led. Low-quality development patterns follow three pathways, which can be summarized into two types: “digital technology–digital environment” constrained and “digital technology–digital organization–digital environment” multi-dimensional constrained. Multi-period comparative analysis reveals multiple trajectory evolution phenomena in the digital economy’s drive toward high-quality development of the smart health and aging industry. Among these, the digital technology-driven and dual-core “digital technology-digital environment” models represent dominant trajectories, while the digital environment-led model constitutes a turning-point trajectory.

**Discussion:**

The digital economy drives the high-quality development of the smart health and aging care industry through multiple pathways and evolutionary trajectories, with the three elements—digital technology, digital environment, and digital organization, functioning synergistically. This research provides theoretical references and practical insights for understanding how the digital economy era propels the high-quality development of the smart health and aging care industry.

## 1 Introduction

The global population structure is undergoing an irreversible trend of aging. Among the world’s most rapidly aging nations with substantial senior populations, China faces particularly severe pressure to transform its older adults care service system. The Seventh National Population Census indicates a population of 260 million aged 60 or older, constituting 18.7% of the overall population (1). This demographic exhibits complex characteristics marked by the overlapping trends of advanced age, empty-nest households, and disability (2). Given this context, failings in conventional senior care infrastructure, including regional imbalances, inefficient resource allocation, and supply–demand imbalances (3, 4), have become increasingly apparent, neglecting the varied and hierarchical health and care requirements of the older adults (5, 6). Consequently, advancing the older adults care industry toward intelligent, advanced progress now stands fundamental to realizing the objectives of “Healthy China 2030” (7). Currently, the global digital economy is undergoing a critical transition from rapid expansion to high-quality development (8). According to World Bank statistics, the global digital economy surpassed $41.5 trillion in 2022, accounting for over 50% of GDP (9). As a catalyst for economic expansion and industrial evolution, the digital economy has been widely applied in smart older adults care sectors such as digital healthcare, remote medical services, and digital health interventions in countries like the US and the EU (10), introducing innovative avenues for the evolution and enhancement of senior care sectors.

Amid the convergence of healthy aging and digital economy trends, the Chinese government has keenly seized development opportunities. It has successively issued policy documents including the action plan for the development of the smart health and aging care industry (2021–2025), the 14th five-year plan for digital economy development, and the opinions on developing the silver economy to enhance the well-being of the older adults (11–13). These endeavors seek to enhance the interconnection between the digital and real economies, fostering innovative commercial paradigms in the smart health and aging care sector (14, 15). In this process, the data-centric economic model driven by digital technology has gradually become a vital force propelling the development and transformation of the smart health and aging care industry. The question of how to tap into the digital economy’s innovative potential and harness its synergistic forces to fuel top-tier growth has become a hot topic among scholars and industry leaders alike (16, 17).

Although existing research has confirmed the positive impact of the digital economy on the development of the smart aging care industry (18, 19), two significant shortcomings remain in this field. On the one hand, the perspectives of current studies are relatively fragmented. Most studies focus on analyzing the application effects of individual technologies like IoT and big data within the aging care industry (20, 21), lacking a systemic integration perspective to deeply explore the synergistic driving mechanisms of different dimensions within the digital economy for the development of the aging care industry (22). On the other hand, the research process exhibits pronounced static characteristics. Existing literature generally overlooks the spatial–temporal heterogeneity and dynamic evolution of the digital economy’s driving effects (23, 24). These studies neither systematically explain the regional differences in smart health and aging care development between China’s eastern and central-western regions across different periods, nor fully reveal the evolving connections and interaction pathways between the digital economy and the older adults care industry over time.

To address the aforementioned research gaps, this research utilizes the Technology-Organization-Environment (TOE) framework and applies fuzzy set Qualitative Comparative Analysis (fsQCA) to systematically explore the complex causal mechanisms through which the digital economy drives high-quality development in the smart health and aging care industry. The TOE framework was selected because it provides an integrated theoretical lens for analyzing technology adoption and digital transformation at the industrial level (25). It systematically accommodates the three core conditions—technology, organization, and environment which align precisely with the systemic characteristics of the smart health and aging care industry, where multiple elements are interwoven. The QCA method was chosen for its high compatibility with the TOE framework: grounded in configurational thinking and set theory, QCA identifies multiple concurrent causal pathways or “different paths leading to the same destination” that produce outcomes. This perfectly aligns with the TOE framework’s emphasis on the intrinsic logic of “multi-factor synergistic interactions,” addressing the limitations of traditional regression methods in capturing such complex, nonlinear interrelationships. Furthermore, the dynamic QCA employed in this study can further reveal the evolutionary trajectories of these effective configurations over time, thereby responding to the academic community’s call for attention to the dynamic nature of digital economic development.

Based on this, this research seeks to examine the following queries: Within the TOE framework, are digital economy factors necessary conditions for driving the high-quality development of the smart health aging industry? Which combinations of digital economy factors can drive the high-quality development of the smart health aging industry? Do equivalent and timely combinations exist among different factor combinations? What are their dynamic evolution trajectories? What roles do digital technology, digital organization, and digital environment, respectively, play in promoting the high-quality development of the smart health aging industry? The marginal contributions of this study are as follows: First, by exploring the patterns and mechanisms through which complex digital economy factors drive the high-quality development of the smart health aging industry using the TOE framework, it expands the theoretical perspective of existing research. Second, the application of the aggregated dynamic QCA method to systematically identify multiple concurrent causal pathways and their evolutionary patterns offers a novel methodological perspective. Third, by revealing the synergistic mechanisms of digital economy factors, it provides empirical evidence for formulating differentiated development policies for the smart health industry.

## 2 Literature review and theoretical framework

### 2.1 Literature review

The integration and development of the digital economy and the health and senior care industry has gradually become a focus of attention for academics and policy makers. While traditional research has mostly focused on the contradiction between supply and demand of senior care services or the single technical application of digital economy, the latest research has emphasized more on the systemic role of digital economy in promoting the high-quality development of the smart healthy aging industry (4). The smart health and aging industry refers to an emerging industrial cluster driven by next-generation information technologies such as the internet of things, big data, artificial intelligence, and cloud computing. Its primary aim serves the older adults requirements in areas such as assistance with daily activities, health management, medical rehabilitation, and mental and cultural well-being (26). At its essence, it represents the deep integration of traditional older adults care services with strategic emerging industries. The high-quality development of the smart healthy aging industry is not only reflected in the improvement of service efficiency, but also in its ability to achieve sustainable, inclusive and precise aging service provision (3). Therefore, more and more scholars have adopted industrial effectiveness as an indicator of the quality of the development of the senior care industry to reflect the degree of optimization of resource inputs and service outputs (15). The facilitating effect of the digital economy on the smart health and aging care industry has been verified in several countries and regions. For example, a study based on a panel data analysis of OECD countries found that the digital economy significantly improved the accessibility of senior care services, especially in the areas of telemedicine and smart care (27). Similarly, using Chinese provincial data, one study demonstrated that the digital economy is significantly and positively related to the efficiency of the smart health and aging care industry, but this relationship is significantly differentiated between eastern and central and western regions (15, 18). This regional disparity has also drawn attention from other scholars. For instance, some studies indicate that while digital technologies can enhance the distribution of aging care resources, weak digital infrastructure may further delay the development of the aging care industry in underdeveloped regions (28).

The impact of the digital economy on the development of the smart health and aging care industry can be explained from three key perspectives. First, the extensive deployment of digital technologies, including the Internet of Things, AI, and cloud computing, can enhance the intelligence of senior care services. For example, smart wearables enable continuous health surveillance for seniors, mitigating critical health incident risks (20, 21), and big data analysis can optimize the allocation of resources in senior care and medical institutions and reduce service redundancy (29). Second, the digital economy has given rise to new forms of business such as “Internet + aging care” and “shared aging care,” which have broken the time and space limitations of traditional aging care services. For example, online consultation platforms have made it possible for older people in remote areas to have access to high-quality medical resources, while the sharing economy model has increased the utilization rate of home beds for the older adults (30). Finally, the government’s digital governance capabilities and industrial policies are crucial to the development of smart healthy aging. For example, studies have pointed out that China’s smart healthy aging action plan effectively promotes the digital transformation of enterprises through financial subsidies and standardization, while the European Union’s digital health strategy promotes cross-border collaboration in older adults care services through data-sharing regulations (31). However, the role of the digital economy in advancing the smart health and aging care industry is not a linear relationship. Some scholars point out that significant disparities exist across regions in terms of digital infrastructure development, levels of digital economic advancement, and governmental digital governance capabilities (24). All of these factors may constrain the high-quality development of the digital economy-driven smart health and aging industry.

In summary, there are still obvious limitations in the theoretical perspectives of studies on the digital economy and the development of the smart health and aging industry. First, although scholars have identified key drivers such as digital infrastructure, policy support, and human capital (15, 24, 31), these studies have mostly focused on the common features of different regions while ignoring the key differences between cases. For example, why has the smart aging industry developed significantly faster in the Yangtze River Delta than in other regions? Why is it difficult for some central and western provinces to realize industrial upgrading despite the same policy support? These unanswered questions highlight the inadequacy of existing studies in providing systematic explanations, and relevant research findings are still characterized by fragmentation (19). Second, existing studies have paid insufficient attention to the dynamic adjustment mechanism of the synergistic development of the digital economy and the smart pension industry. This limitation makes it difficult for researchers to adequately explain the formation mechanism of the health digital divide (24), and to dissect rebound effects such as the mismatch between technology application and aging-appropriate demand (28). Much of this theoretical blind spot stems from the fact that most studies have focused excessively on the independent effects of a single factor while ignoring the interactions between system elements. Third, existing studies have relied heavily on linear regression models to estimate the net effect of specific variables (16, 18, 24). Although this approach can identify the key influencing factors, it is difficult to capture the complex causal relationship between the digital economy and the development of the senior care industry, especially the phenomenon of “different paths to the same destination” in different development paths. In addition, there are obvious limitations in the traditional measurement methods in dealing with the problems of multicollinearity and variable selection bias (32), which to a certain extent reduces the robustness of the research conclusions. Hence, the evolution of the tech-fueled smart health and aging sector is typically shaped by a multitude of variables, including technological advancements, organization and environment, the adoption of a group perspective may be more suitable for exploring the complex mechanisms between the two.

### 2.2 Theoretical framework

To elucidate the mechanism by which the digital economy fosters the advancement of the smart, healthy aging sector, this study adopts the TOE framework for analysis. The TOE framework, as a comprehensive analysis of technology adoption and innovation models, emphasizes the combined impact of various elements indicated from the three dimensions of technology, organization, and environment (25). Among them, technological conditions focus on fitness and benefits, organizational conditions contain internal characteristics such as scale and capacity, and environmental conditions involve external factors such as policies and markets. This framework is widely used in the field of economic management and has strong explanatory power when dissecting the interaction effects between its technology, organization and environment (33). Similarly, digital economy-driven high-quality development of the smart health and aging care industry also relies on the coupling and synergy of the three, which is highly compatible with the TOE theoretical framework. The logical basis for the development of smart health and aging care industry driven by digital economy is as follows:

#### 2.2.1 Digital technology

Digital technology is the core support for driving the high-quality development of smart health and aging care industry, which mainly covers the dimensions of digital infrastructure and digital technology application. The synergistic effect of these two elements can significantly improve the accuracy and accessibility of senior care services and promote the digital transformation of the senior care industry (34). On the one hand, digital infrastructure provides key support for the development of the smart aging care industry. The hardware system constituted by the Internet of Things, 5G networks and smart terminal devices, together with the software system formed by the health big data platform and cloud computing center, builds the technological base for the development of the smart aging care industry (21, 34). The digital infrastructure makes remote medical diagnosis more convenient and the data transmission of smart devices more stable, helping precise health management. At the same time, cloud computing helps the integration and efficient deployment of resources for senior care services, and the Internet of Things realizes the intelligent monitoring of the senior care environment, which promotes the development of the smart health and aging care industry in an all-round way (20, 35). On the other hand, the application of digital technology provides a strong impetus for the development of the smart aging care industry. With the increasing integration of AI, big data, and other digital technologies in conventional elder care systems, for example, telemedicine technology breaks the geographical limitations to realize at home medical treatment, and smart wearable devices monitor health data in real time (36, 37). These technological applications not only optimize the service process, but also achieve the precise allocation of resources in the senior care industry through data-driven (38).

#### 2.2.2 Digital organization

Digital organization is a key element to promote the high-quality development of the smart healthy aging industry, covering the dimensions of digital organization innovation and digital human capital. Digital organizational innovation reconfigures the ecology of the smart healthy aging industry through intelligent management and promotes its high-quality development. One aspect is digital organizational innovation improves the operational efficiency of the industry by optimizing the business processes and resource allocation of the smart healthy aging industry (39). By integrating data from multiple parties such as senior care service organizations and medical units, it breaks down information silos and realizes data sharing and collaborative work (40). With the help of intelligent algorithms, it can accurately match the needs of the older adults with service resources, optimize the service process, and improve the speed and quality of service response (41). At the same time, the digital organization can also conduct real-time monitoring and evaluation of industrial operations, help enterprises make scientific decisions, and promote the standardization and intelligent development of the intelligent healthy aging industry (42, 43). On the other hand, Digital human capital fosters the refined growth of the intelligent, healthy aging sector via intelligent knowledge expertise. Digital professionals master digital technology and are able to accurately use smart devices to monitor health, analyze data, and provide personalized senior care services (28). At the same time, enhancing practitioners’ digital skills through online training can broaden the boundaries of the development of the senior care industry (44). Digital manpower can also optimize industrial management, help accurate decision-making with the help of data analysis, attract investment, promote industrial innovation and upgrading, improve service quality and operational efficiency, and help the smart health and aging care industry flourish (28).

#### 2.2.3 Digital environment

The digital environment conditions cover the two dimensions of government digital policy and digital financial inclusion. Government digital policy accelerates the high-quality development of the smart health and aging care industry through top-level design and financial support standardization (45). Government digital policies optimize application scenarios such as health monitoring and telemedicine by promoting new infrastructures such as 5G, IoT, and big data to cover the senior care sector (46). At the same time, it promotes the development of the smart aging industry through financial subsidies and pilot demonstrations to reduce the cost of enterprise innovation, data sharing and security regulations to promote the integration of medical and nursing resources (47), and the development of new projects in the aging industry (48, 49). Digital inclusive finance helps upgrade the smart health care industry through digital inclusive finance by lowering the financing threshold and innovating payment methods (50). Digital financial technology provides convenient credit for small and medium-sized senior care enterprises, lowers the cost eligibility for technology-enabled older adults care services, and promotes the research and development of intelligent equipment and age-appropriate transformation (51). Meanwhile, mobile payment and insurance technology optimize the settlement of pension expenses and health risk management, and block chain technology guarantees the safe circulation of pension and medical data (16). Digital inclusive finance expands the coverage of services so that more older adults can enjoy affordable smart care, remote consultation, and other digital pension services (52), thereby promoting the long-term sustainable development of the aging care industry.

**Figure 1:**
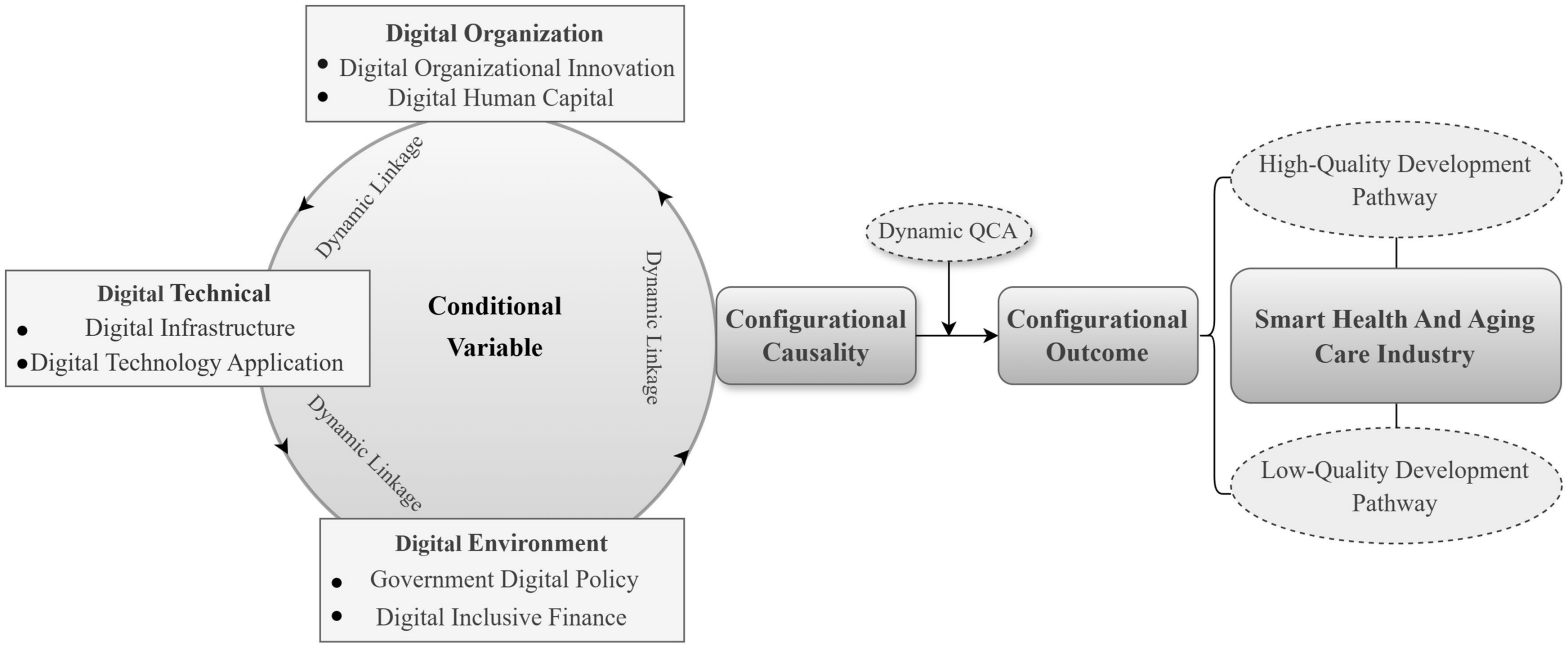
Theoretical analysis framework.

Based on this, the research establishes a theoretical analytical framework for the high-quality development of the smart health and aging care industry driven by the digital economy based on the TOE framework, which is proposed to be constructed from three dimensions and six sub-conditions, namely, digital technology, digital organization, and digital environment. These dimensions include digital infrastructure and digital technology application under the digital technology condition; digital organizational innovation and digital human capital under the digital organization condition; and government digital policy and digital inclusive finance under the digital environment. The framework systematically explains the intrinsic mechanism of the digital economy to drive the high-quality development of the smart health and aging care industry. As shown in Figure 1.

## 3 Research design

### 3.1 Research methods

**Figure 2:**
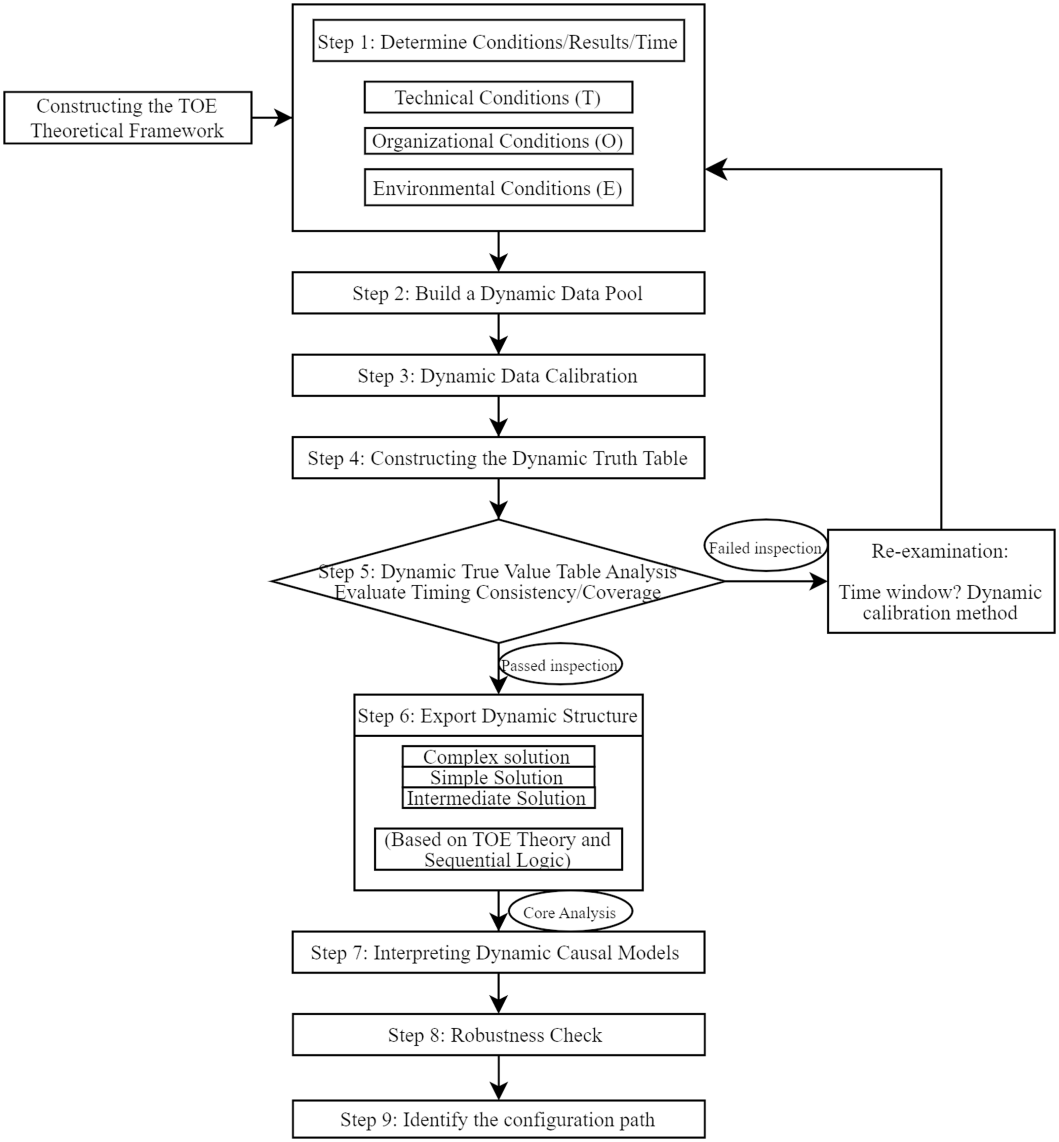
Dynamic QCA implementation process.

To systematically analyze the dynamic causal mechanisms by which the digital economy drives high-quality development in the smart health and aging care industry, this study employs the Dynamic QCA method. Proposed by García-Castro and Ariño, this approach effectively integrates panel data with configuration comparison logic, overcoming the limitations of traditional QCA in analyzing temporal dimensions (53). Its implementation involves three primary steps: First, data calibration and configuration construction. Based on theoretical frameworks and data distribution, all condition variables and outcome variables from the provincial panel data (cases) spanning 2012–2022 are uniformly calibrated into fuzzy set scores. Second, dynamic analysis operations: the resulting “case-year” configuration data is imported into the R programming environment. By calculating three core metrics aggregate consistency, inter-group consistency, and intra-group consistency the robustness of configurations is systematically evaluated at the overall, cross-case, and temporal levels. Finally, configuration evolution trajectory identification: Based on the above analysis results, key configuration paths were identified through multi-period comparisons. Their dominant, complementary, or transitional evolution trajectories were extracted based on their emergence and succession across different time periods. Compared to traditional QCA, dynamic QCA employs a multi-level consistency analysis framework. This approach not only identifies equivalent paths that converge to the same outcome but also precisely quantifies the dynamic characteristics of these paths as they vary over time and across cases. Consequently, it reveals the causal complexity within the temporal dimension more profoundly. Based on the TOE framework, this study constructs a standard implementation flowchart for dynamic QCA, as shown in Figure 2.

### 3.2 Research data and variable measurement

#### 3.2.1 Sample data

The study examines 30 Chinese provinces (excluding Tibet, Hong Kong, Macao, and Taiwan) as the sample area. Meanwhile, considering that China began to implement smart aging in 2012, this study selects inter-provincial panel data from 2012 to 2022, which effectively overcomes the static limitations of traditional cross-sectional data and significantly improves the study’s dynamic explanatory power and empirical accuracy through the integration and standardization of data in the spatio-temporal dimension. The data sources include: First, the data on core indicators of the development of the smart health and aging care industry: they are mainly derived from the China statistical yearbook, the China population and employment statistical yearbook, the China social statistical yearbook, the China labor statistical yearbook, the China tertiary industry statistical yearbook, as well as the statistical yearbooks, official websites, and statistical bulletins of each province. Secondly, the data reflecting the level of digital economy mainly come from China science and technology statistical yearbook, China information yearbook, China statistical yearbook and provincial statistical yearbooks. At the same time, the data were collected and organized using the CEIC data network, and the missing data were filled in reasonably. Among them, the word frequency of digital economy policy in the government work report comes from text mining analysis technology, and the financial digitization index data originates from the Peking university digital inclusive finance Index, as compiled by the Peking university digital research center, as shown in Table 1.

**Table 1:** Description of variable indicators.

Level 1 indicators			Level 2 indicators	Indicator description
Conditional variable	Digital technical	Digital infrastructure	Internet penetration rate	Number of Internet users as a proportion of resident population (%)
			Telephone penetration rate	Total number of telephones (including mobile phones)/total population in administrative area*100 (units)
			Length of long-haul fibre-optic cable lines	Length of long-haul fibre-optic cable lines (10,000 km)
			Internet broadband access ports	Number of Internet broadband access ports (10,000)
			Number of Internet domain names	Number of Internet domain names (10,000)
		Digital technology application	Volume of telecommunication services	Total telecommunication services per capita
			Software business volume	Software business income per capita
	Digital organizational	Digital organizational innovation	Number of new product development projects	Number of new product development projects per capita in the region in the current year
			Expenditure on new product development	Per capita expenditure on new product development in the region in the current year
			Revenue from sales of new products	Revenue from sales of new products per capita in the region in the current year
			Patents granted for inventions	Effective patents for inventions per 100 persons in the region in the current year
		Digital human capital	Digital economy talent	Percentage of employees in computer services and software
			Digital research talent	Total R&D staff for the year
	Digital environmental	Government digital policy	Digital government digital economy focus	Frequency of occurrence of keywords of digital economy in the government work report of that year
			Financial support for science, technology and innovation	R&D expenditure per capita for the year
		Digital inclusive finance	Digital inclusion financial digitisation index	Peking University Digital Finance Inclusion Index
Outcome variable	High-quality development of the smart health and aging care industry	Innovative development	the smart health and aging care industry innovation strength	Internal expenditure on R&D/GDP
			the smart health and aging care industry	R&D personnel converted to full-time equivalents
			Patent scale of the smart health and aging care industry	Total number of patents granted/resident population of the region
			Patent quality in the smart health and older adults care industry	Number of patents granted for inventions/total number of patents granted
		Co-ordinated development	Difference between urban and rural consumption	Per capita consumption expenditure of urban residents/per capita consumption expenditure of rural residents
			Urban/rural income differentials	Urban disposable income per capita/rural disposable income per capita
			General public budget consumption expenditure on old age than	General public budget pension expenditure/population
			Percentage of fiscal expenditure on older adults	Fiscal expenditure on old-age pensions/fiscal expenditure
		Shared development	Pension insurance coverage	Number of persons enrolled in basic old-age insurance/number of persons liable to be enrolled
			Pension coverage	Number of socialised pensioners/population aged 55 and over
			Number of beds per 1,000 older adults	Number of beds for the older adult aged 65 and over (in thousands)
			Medical insurance participation rate	Number of persons enrolled in basic health insurance/total number of persons
			Health technicians per 1,000 population	Number of health technicians/population (thousands)
			Number of community services and facilities per 10,000 population	Number of community services and facilities/population (10,000)
			Qualified social workers per 1,000 older adults	Cumulative number of qualified social workers/population aged 65 and over (in thousands)
		Sustainable development	Older adults dependency ratio	Number of persons aged 65 and over/number of persons aged 15–64 in the labour force
			Labour force literacy	Average years of schooling of the population aged 15 years and over
			Internet penetration	Internet broadband subscribers/population
			Primary care load	Number of people aged 65 and over/number of primary care facilities
		Efficient development	Concentration of fixed asset investment in the smart health and aging care industry	Investment in fixed assets in industry/investment in fixed assets in the tertiary sector
			the smart health and aging care industry employee density	Number of persons employed in industry/population
			the smart health and aging care industry capital productivity	Industrial value added/industrial fixed asset investment
			Labour productivity in the smart health care industry	Value added of industry/number of people employed in industry
			GDP contribution of the smart health and older adults care industry	Incremental industrial value added/GDP

#### 3.2.2 Variable measurement

##### 3.2.2.1 Conditional measurement

Digital Infrastructure, with reference to Hu et al. Five basic indicators, namely, Internet penetration rate, telephone penetration rate, long-distance fiber optic cable fiber length, Internet broadband access ports, and the number of Internet domain names, were selected to construct a composite index through entropy method to thoroughly evaluate digital resource availability (54). In order to eliminate subjective assignment bias, all the composite indexes in this study are measured by the entropy method. Digital technology application, drawing on Liu and Xu, is used to construct a composite index based on 2 core indicators: telecommunications business volume and software business volume (19).

Digital Organizational Innovation, based on the idea of Tsou and Che (55), select the number of new product development, new product development expenditure, new product sales revenue, invention patent authorization 4 core indicators to construct a comprehensive assessment index. Digital human capital draws upon the methodology described by Gong et al. (56), and adopts 2 core indicators, namely, the proportion of IT professionals and the overall count of R&D staff for the present year, for the development of a holistic evaluation metric.

Government Digital Policy, referring to Gan et al. (57), constructs a comprehensive assessment index based on 2 core indicators: total number of keyword word frequencies of digital economy in the government work report and per capita R&D expenditure. Digital financial inclusion, referring to Yu et al. (58), adopts the financial digitization index as a metric to measure digital financial inclusion.

##### 3.2.2.2 Outcome measurement

High quality development of the smart health and aging care industry. Citing the methodology employed by Wei et al. (23), five secondary indicators of innovative development, co-ordinated development, shared development, sustainable development, and efficient development and 24 tertiary indicators are used to characterize the level of high-quality development of the smart health and aging care industry, and the evaluation results of the indexes are comprehensively measured applying the entropy model as a universal gauge to assess the advancement standard of the smart aging industry. All variable indicators and their measurements are shown in Table 1.

### 3.3 Variable calibration

Drawing on existing literature, the data were standardized in this study. Based on the sample and variable distribution characteristics, the internal direct calibration method of fsQCA was used to convert the raw observations into affiliation scores in the (0–1) interval (53). Three key anchor points were set for the specific calibration process: the 95th quartile (full affiliation), the 50th quartile (crossover point), and the 5th quartile (no affiliation at all) (53). In order to avoid omission of data at intersection point 0.5 in the truth table analysis, a fine-tuning treatment of 0.001 was applied to the 0.5 variable values during the preprocessing stage. The specific calibration results are shown in Table 2.

**Table 2:** Variable calibration results.

Variable classification	Variable name		Full affiliation	Junction	Totally unaffiliated
Outcome variable	High-quality Development of the smart health and aging care industry Aging Industry		0.543	0.414	0.324
Conditional variable	Digital technical	Digital infrastructure	0.352	0.097	0.023
		Digital technology application	0.157	0.030	0.003
	Digital organizational	Digital organizational innovation	0.290	0.042	0.008
		Digital human capital	0.392	0.075	0.016
	Digital environmental	Government digital policy	0.339	0.133	0.026
		Digital inclusive finance	398.597	267.950	99.041

## 4 Results

### 4.1 Individual conditional necessity analysis

This study follows the principle of necessity test of the standard QCA method, whereby when the consistency coefficient of a conditional variable exceeds the critical threshold of 0.9, it can be determined that the variable constitutes a necessary condition for the outcome variable (59). Under the dynamic QCA analysis method, two key indicators, inter-group consistency adjustment distance and inter-group coverage, need to be additionally examined. Specifically: first, when the adjustment distance is less than 0.2, it indicates that the aggregated results have a high degree of accuracy, which can be directly used as the basis for the judgment of necessity; second, when the adjustment distance is more than 0.2, it is necessary to validate its necessity through a supplemental test (53). The results of the analysis of the necessity conditions for the high/low quality development of the smart health and aging care industry (as shown in Table 3), specifically, the aggregated consistency level of the six antecedent condition variables is less than 0.9, indicating that these variables are not the necessity conditions for the outcome variables. Moreover, by observing the inter-group consistency adjustment distance of the 12 types of the 6 conditional variables, it is found that the inter-group consistency adjustment distance of either the high/low quality development of the smart health and aging care industry is less than 0.2. Therefore, there is no necessary condition that constitutes the outcome variable. Taken together, none of the six single conditional variables is a necessary condition leading to the high/low quality development of the smart health and aging care industry. This means that the digital economy-driven development of the smart health and aging care industry is not the net utility of a single factor, but is subject to the complex linkage and synergistic matching of multiple factors.

**Table 3:** Results of the analysis of the conditions of necessity.

Conditional variable	High quality development of the smart health and aging care industry				Low quality development of the smart health and aging care industry			
	Aggregation Consistency	Summary Coverage	Intergroup consistency adjustment distance	Intra-group consistency adjustment distance	Aggregation Consistency	Summary Coverage	Intergroup consistency adjustment distance	Intra-group consistency adjustment distance
Digital infrastructure	0.785	0.775	0.029	0.058	0.623	0.505	0.060	0.075
~Digital infrastructure	0.498	0.617	0.141	0.083	0.722	0.733	0.040	0.064
Digital technology application	0.864	0.812	0.023	0.038	0.596	0.459	0.075	0.057
~Digital technology application	0.424	0.561	0.188	0.093	0.756	0.820	0.051	0.051
Digital organizational innovation	0.772	0.749	0.014	0.052	0.649	0.516	0.043	0.07
~Digital organizational innovation	0.502	0.635	0.112	0.087	0.685	0.711	0.015	0.074
Digital human capital	0.725	0.692	0.033	0.062	0.710	0.555	0.031	0.07
~Digital human capital	0.534	0.692	0.064	0.091	0.606	0.644	0.024	0.097
Government digital policy	0.765	0.784	0.037	0.049	0.590	0.496	0.085	0.07
~Government digital policy	0.508	0.602	0.139	0.071	0.742	0.722	0.050	0.051
Digital inclusive finance	0.811	0.905	0.077	0.021	0.459	0.420	0.156	0.042
~Digital inclusive finance	0.480	0.520	0.176	0.043	0.896	0.796	0.084	0.019

### 4.2 Conditional grouping sufficiency analysis

Conditional grouping sufficiency analysis was further employed to reveal the effect of multivariate synergy on the outcome. Citing the research methodologies employed by earlier scholars (60), the consistency threshold condition is used to determine whether there is sufficiency between the condition and outcome variables. Specifically, the established analysis criteria include a consistency level for group path at 0.8, a threshold for PRI at 0.7, and a minimum case occurrence of 1. The ensemble solution is obtained through R language computation, and intermediate solution is the main solution, and parsimonious solution is the auxiliary solution for validation, which identifies the core and auxiliary conditions of the group path. Considering the asymmetry of the role of factors, the independent group state analysis of high/low quality development of the smart health and aging care industry is carried out respectively, and a total of 8 group state paths for the high-quality development of the smart health and aging care industry are obtained. The detailed outcomes are presented in Table 4.

**Table 4:** Results of high/non-high-quality development grouping analysis of the smart health and aging care industry.

Conditional variable	High quality development of the smart health and aging care industry					Low quality development of the smart health and aging care industry		
	Digital technology-driven	Digital technology–digital environment dual core propulsion			Digital environment leadership	Digital technology–digital environment co-bound type	Digital technology–digital organisation - digital environment multidimensional constraints type	
	M1	M2	M3	M4	M5	M6	M7	M8
Digital infrastructure	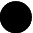	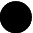	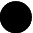				⨂	
Digital technology application	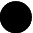	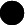	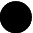	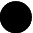		⨂	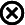	⨂
Digital organizational innovation	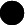	⨂	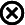	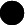	⨂		⨂	⨂
Digital human capital	⨂		⨂			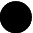		
Government digital policy	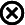		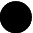	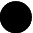	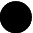			⨂
Digital inclusive finance		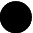		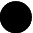	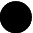	⨂	⨂	⨂
Consistency	0.933	0.939	0.934	0.942	0.917	0.923	0.896	0.897
PRI	0.836	0.899	0.855	0.904	0.811	0.788	0.771	0.773
Degree of coverage	0.347	0.686	0.376	0.674	0.413	0.507	0.554	0.560
Unique coverage	0.008	0.020	0.007	0.053	0.008	0.118	0.011	0.010
Inter-group consistency adjustment distance	0.011	0.012	0.027	0.016	0.023	0.018	0.028	0.027
Intra-group consistency adjustment distance	0.029	0.029	0.029	0.036	0.030	0.019	0.026	0.026
Overall PRI	0.819					0.897		
Overall consistency	0.888					0.773		
Overall coverage	0.810					0.560		

Large 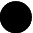 and 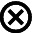 indicate the presence or absence of core conditions, small 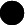 and 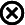 indicates the presence or absence of an auxiliary condition; a blank space indicates that presence or absence is also possible.

#### 4.2.1 Summary results

From Table 4, the overall consistency ratios of the solutions are evident of the high-quality development and low-quality development of the the smart health and aging care industry are 0.888 and 0.773, respectively, which are higher than the standard threshold of 0.75, and the overall coverage is greater than 0.5. Subsequent assessments revealed the distances for adjusting inter-group and intra-group consistency metrics of all the groupings are all at a distance of less than 0.2, which suggests that the grouping paths have a higher explanatory effectiveness. Based on the cluster analysis of the grouping features, five development patterns were finally identified. Among them, three high-quality development modes are digital environment-led, “digital technology-environment” dual-core driven and digital technology-driven; and three low-quality development modes are “digital technology-environment” constrained and “digital technology-organization-environment” constrained. The low-quality development model includes the “digital technology-environment” constraint type and the “digital technology-organization-environment” multi-dimensional constraint type.

##### 4.2.1.1 Analysis of high grouping results

First, digital technology-driven (M1), with a grouping consistency of 0.933 and coverage of 0.347, explains 34.7% of the sample cases. In the M1 grouping pattern, the core conditions are digital infrastructure and digital technology application, and the auxiliary condition is digital organizational innovation. Even if digital human capital is insufficient, digital infrastructure and digital technology application are fully developed and digital organizational innovation is carried out to assist, it can drive the high-quality development of the smart health and aging care industry. Typical cases include Anhui Province and Fujian Province. Under this model, the digital economy drives industrial development from two dimensions: digital infrastructure and digital technology application. On the one hand, digital infrastructure such as cloud computing, data centers, IoT and communication networks help senior care institutions to achieve service docking with healthcare institutions and families, efficiently process senior care service data, and reduce operating costs. On the other hand, digital technology applications are connected to senior care service platforms to monitor the health status of the older adults in real time, provide accurate services, and improve service quality, management level and allocation efficiency. Taking Anhui Province as an example, its path selection is rooted in its resource endowments and development strategy: As a pioneer zone for digital infrastructure in central China, Anhui possesses first-mover advantages in communication networks and computing resources. By 2024, the province had cumulatively built 140,000 5G base stations and established China’s first domestically developed computing cluster with 10,000 cards and 3,000 Pflops of computing power. It has essentially constructed a province-wide smart aging care service information network and data sharing platform. This infrastructure advantage provides the material foundation for its technology-driven path, enabling it to prioritize data integration and platform-based operations. This approach accelerates the convergence of digital technologies like big data and artificial intelligence within aging care scenarios, thereby fostering new business models and creating demonstration effects. Compared to other regions, Anhui faces relative weaknesses in social organization capacity, high-end talent reserves, and financial service systems. These constraints collectively preclude the province from pursuing talent-driven or finance-driven development paths. Consequently, it has opted for a strategy centered on “hard technology” and “robust infrastructure” as breakthrough points to compensate for its relative deficiencies in human capital and financial resources. This resource structure disparity has prompted Anhui to strengthen government guidance and infrastructure investment, forming a technology-centric industrial development logic. This validates how resource endowments shape development path choices. Unlike provinces relying on robust fiscal subsidies for inclusive finance or leveraging top-tier university resources to cultivate human capital, Anhui’s path demonstrates the logic of “not optimal but best-fit” development. Accordingly, the government’s role has centered on guiding and investing in infrastructure development rather than directly intervening in organizational transformation or financial markets. This has ultimately forged a distinct development chain of “technology empowerment—business model innovation.” This case demonstrates that high-quality development in regional smart health and older adults care industries does not follow a single paradigm but emerges as a differentiated path choice under specific resource constraints.

Second, the “digital technology-digital environment” dual-core driving type (M2, M3, M4), the consistency of the grouping is 0.939, 0.934, 0.942, the coverage is 0.686, 0.376, 0.674, which can explain 68.6, 37.6, 67.4% of the sample cases. Grouping M2 takes digital infrastructure and digital inclusive finance as the core conditions, which suggests that the interactive integration of digital infrastructure and digital inclusive finance can promote the high-quality development of the smart health and aging care industry when digital organizational innovation is insufficient; Grouping M3 takes digital infrastructure, digital technology application, and governmental digital policy as the core conditions, which suggests that in the case of the absence of a core of digital human capital and the absence of digital organizational innovation is missing, the digital economy through digital infrastructure, digital technology application and government digital policy can also promote the high-quality development of the smart health and aging care industry; M4 with digital technology application, government digital policy and digital inclusive finance as the core conditions, this grouping shows that through the play of the digital environment, such as the government digital policy and digital inclusive finance, as well as synergistic effect with the application of digital technology, it also can promote the high-quality development of the smart health and aging care industry. Taken together, the M2, M3 and M4 groupings reflect the driving effect of the “digital technology-digital environment” dual-core promotion on the high-quality development of the smart healthy aging industry. It can be seen that local governments can provide sufficient resources for the senior care industry through the integration of digital technology and digital external environment resources, and then effectively drive the high-quality development of the smart health and aging care industry. Typical cases include Zhejiang Province, Shandong Province and Tianjin City, which have a better foundation for economic development and superior resource endowments. Taking Zhejiang Province as an example, at the technological level, the province systematically advances the deployment of digital information infrastructure. Leveraging its outstanding market-driven application capabilities, it facilitates precise alignment between digital technologies and the aging care industry on both the supply and demand sides. The “Zhejiang Smart Aging Care” platform, spearheaded by the province, not only achieves effective integration of service resources and older adults care data but also progressively fosters an open and inclusive digital service ecosystem. This provides diverse market entities with a unified access point and collaborative innovation environment. At the environmental cultivation level, Zhejiang leverages its inherent advantage of a vibrant private economy. Through policy guidance, it promotes technological collaboration and open patent sharing among smart aging care enterprises, significantly accelerating the transformation and application of emerging technologies in aging care scenarios. Concurrently, local governments have transitioned from direct builders to institutional designers and market cultivators. This is manifested in actively piloting digital financial services for aging care, continuously refining financial service systems for the aging care industry, and effectively guiding social capital participation. These efforts provide robust financial support and market space for the high-quality development of the smart health and aging care industry. Zhejiang’s ability to forge a dual-core driving path of “technology empowerment and institutional synergy” fundamentally stems from the combined effects of its resource endowments and regional development DNA. Compared to regions like Fujian and Anhui, which primarily rely on infrastructure breakthroughs, Zhejiang holds comparative advantages in private economic vitality, financial market maturity, and innovation-entrepreneurship ecosystems. This endowment structure enables it to avoid over reliance on infrastructure investment to compensate for soft capability gaps while sidestepping the developmental challenges faced by resource-scarce areas. A robust market presence, active private capital, and an efficient, open governance system collectively foster a regional development ecosystem that incentivizes technological innovation while accommodating institutional innovation. This ecosystem has organically evolved into a high-quality development path characterized by the coupling of technology and environment, and the synergy between hard and soft power.

Third, digital environment leading (M5), accounts for 41.3% of cases with 0.917 grouping consistency and 0.413 original coverage. In the group state M5, digital financial inclusion and the government digital policy are the core condition variable, indicating that the local government provides financial support for the senior care industry from the dimensions of financing support, technological promotion, and industrial ecological optimization through the introduction of the senior care financial policy and encouraging the digital financial market to use diversified financial instruments, thus promoting the high-quality development of the smart healthy senior care industry. Typical cases include economically developed coastal regions such as Shanghai, Jiangsu Province and Guangdong Province, which, with their strong endowment of industrial capital and financial resources, establish foundational requirements for digital finance advancement in senior care technology. The study found that digital finance in these regions mainly promotes the development of the senior care industry through a dual path: On the one hand, in the supply of funds, by lowering the financing threshold and broadening financing channels, the measure effectively clears up the financial hurdles involved in establishing advanced eldercare facilities and advancing intelligent technology; at the same time, it fosters industry structure by fostering industry coalitions. and comprehensive service platforms, it realizes the efficient allocation of service resources, information elements and financial capital, which in turn promotes the innovative development of the senior care industry. Second, in terms of industrial organization, through the construction of industrial alliances and comprehensive service platforms, the efficient allocation of service resources, information elements and financial capital has been realized, which in turn promotes the development of the senior care industry in the direction of intelligence and efficiency. Taking Shanghai as an example, its development path profoundly reflects the systemic synergy between institutional environment and financial capital. The city has successively formulated and implemented a series of policy documents, including the action plan for promoting the development of aging care technology innovation in Shanghai, strategically establishing a smart aging care development model driven by both technological innovation and inclusive finance. At the practical level, Shanghai has built a multi-tiered, full-cycle digital financial support system: On the one hand, it precisely channels social capital into the older adults care technology sector through targeted credit guidance and interest subsidy policies; on the other hand, it vigorously encourages the progression and utilization of groundbreaking insurance solutions, annuity, and wealth management products tailored for smart aging care scenarios, while establishing government-guided, socially capital-participated investment funds for the aging care industry. These institutional arrangements have not only effectively broadened financing channels but also formed a closed-loop support mechanism for risk mitigation, technology transfer, and business model innovation. Compared to the “technology-environment” driven approach exemplified by Zhejiang, Shanghai’s path selection is rooted in its unique resource endowments and urban functional positioning. As a national financial center and a testing ground for institutional innovation, Shanghai possesses significant advantages in financial institution concentration, capital liquidity, policy autonomy, and international resource connectivity. However, it lacks cost and land advantages in the large-scale construction of physical digital infrastructure. Therefore, Shanghai has not followed the traditional “hardware-first, technology-driven” path. Instead, it leverages its core competitiveness in institutional supply and financial ecosystems. By establishing a high-level environment driven by both policy and finance, it attracts premium market entities, stimulates organizational innovation and technological integration, thereby achieving an overall leap in industrial capabilities. This approach essentially embodies a composite governance model of “institutional empowerment” and “financial leverage,” with its underlying logic manifested in a progressive transmission mechanism: “policy guidance–financial synergy–ecosystem construction–capability enhancement.” This highlights the pivotal role of a high-end institutional environment in reshaping the industrial innovation ecosystem.

##### 4.2.1.2 Analysis of the results of the low grouping state

The low grouping state indicates that the driving force of the digital economy for the high-quality development of the smart health and aging care industry is insufficient, and there may be a blind spot in the implementation. Comparative analysis of the low group states in Table 4 reveals that the lack of core conditions is the main reason for low-quality development.

First, the “digital technology-digital environment” common constraint type (M6), with a group consistency of 0.923 and a coverage of 0.507, can explain 50.7% of the sample cases. The representative cases are Shanxi Province, Hebei Province, and Hainan Province. Under this model, digital technology application and digital inclusive finance are generally lacking and insufficient, and although digital human capital provides auxiliary functions, it needs to be matched with other conditions to efficiently promote the development of the industry, so there is still a large space for the high-quality development of the smart health and aging care industry. The reason why the model has not yet formed a higher level of development of the senior care industry may be due to a combination of multiple factors such as the application of digital technology and the serious lack of development of digital inclusive finance. Taking Shanxi Province as an example, the underlying reason for its relatively low level of smart health and aging care industry development can be attributed to systemic bottlenecks caused by an imbalance in the “technology-finance” dual structure. On the technological front, the region’s digital applications remain largely confined to foundational informatization tasks like institutional management and information registration. There is a lack of deep integration between cutting-edge technologies—such as artificial intelligence, the Internet of Things, and big data—and core older adults care scenarios like personalized care, health prediction, and telemedicine services. This prevents the marginal benefits of technology-driven industrial upgrading from materializing. On the financial front, the digital inclusive finance ecosystem supporting smart aging care remains underdeveloped. This manifests as: a lack of specialized credit support and interest subsidy policies for smart older adults care technology enterprises; insufficient supply of innovative insurance and wealth management products tailored to the older adults demographic, and a severe shortage of early-stage venture capital and industrial funds dedicated to the older adults care sector. Within this structural predicament, even though Shanxi Province has cultivated a reserve of digital professionals, the absence of high-level technology application platforms to anchor these capabilities and the lack of robust financial channels to translate ideas into commercial practice hinder the effective activation and infusion of human capital into the industrial innovation chain. This ultimately constrains the high-quality development of the regional aging care industry.

Second, the “digital technology–digital organization–digital environment” multidimensional restriction type (M7, M8), the consistency of the group state is 0.896, 0.897, the coverage is 0.554, 0.560, can explain 55.4, 56.0% of the sample cases. The representative cases are Guizhou Province, Yunnan Province, Shaanxi Province, Gansu Province and Qinghai Province. In this pathway, the application of digital technologies, digital organizational innovation, and digital inclusive finance collectively form the core missing conditions. This implies that when these three key elements simultaneously remain at low levels or are absent in regional development, a systemic “development bottleneck” emerges. The core logic of this pathway is that even if other variables (such as digital infrastructure or digital government policies) exhibit localized strengths, their positive effects are diluted or neutralized by the collective absence of core driving conditions, making it difficult to translate them into effective momentum for high-quality industrial development. In stark contrast to successful pathways like Shanghai (dual-engine drive of institutions and finance) and Anhui (technology-infrastructure lead), these western regions commonly face the predicament of multiple concurrent constraints: lagging digital technology adoption results in low levels of smart aging care services; insufficient digital organizational innovation makes traditional care institutions ill-equipped for digital transformation; and the absence of digital inclusive finance cuts off the critical capital lifeline for innovation. This triple deficit—technology, organization, and environment forms a mutually reinforcing negative feedback loop that suppresses the emergence of endogenous growth momentum within the industry, thereby constraining the high-quality development of the smart health and aging care industry.

#### 4.2.2 Intra-group results

**Figure 3:**
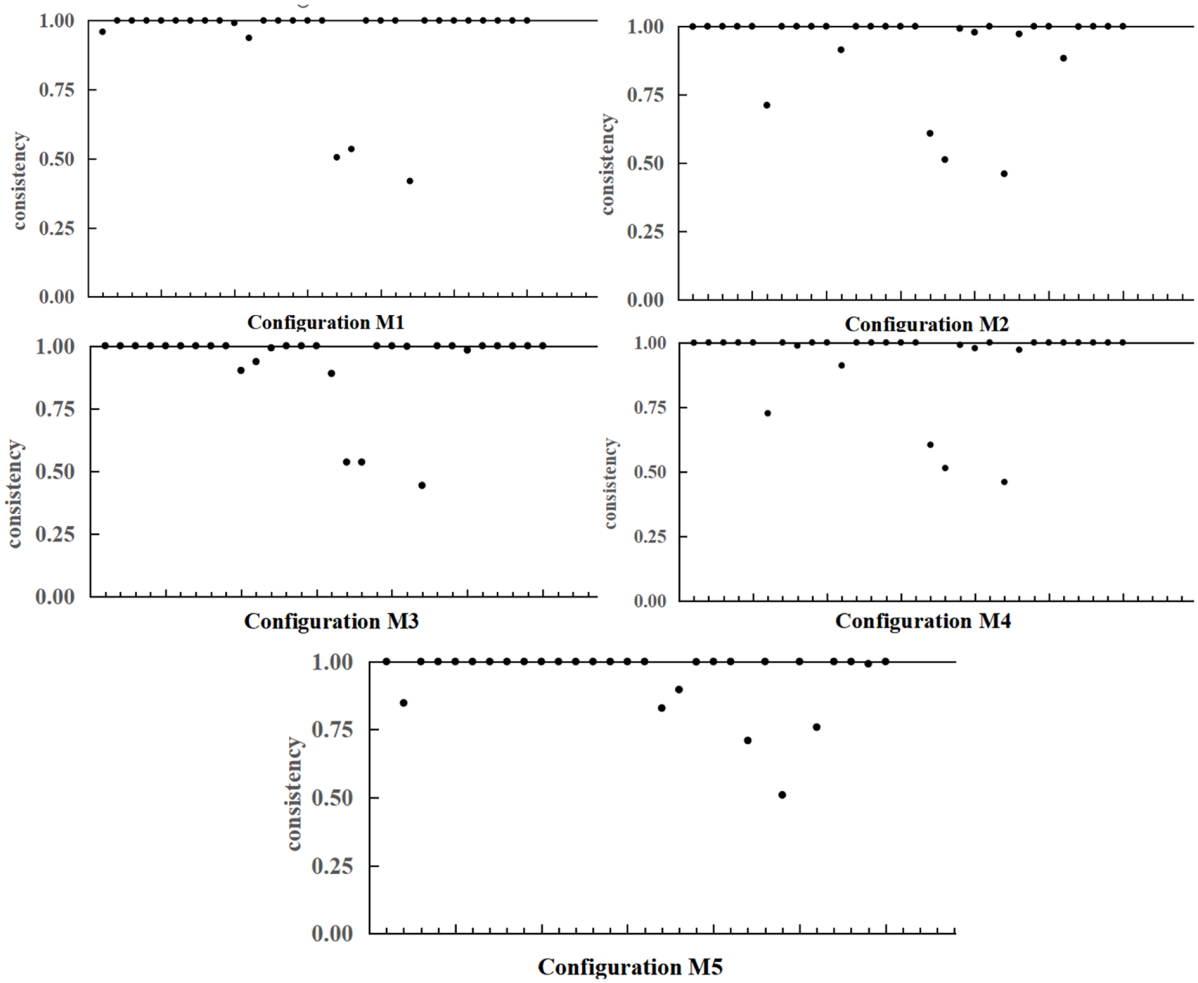
Configuration M1–M5 intra-group consistency.

Among the five high-quality development grouping paths, the intra-group consistency adjustment distance is below the threshold of 0.2, demonstrating that the explanatory validity of each cluster is regionally consistent and exhibits no notable “case” influence. However, there is a gradient difference in the spatial distribution of high-quality development grouping cases: the developed eastern coastal regions such as Shanghai, Jiangsu and Guangdong are in the first gradient; Shandong, Anhui and Fujian are in the second gradient; Hubei, Hunan and Shaanxi are in the third gradient; and Guizhou, Gansu and Ningxia are in the fourth gradient. The high-quality development grouping is mainly concentrated in the eastern and central regions of the nation, while the low-quality development grouping is concentrated in the less economically developed regions in the central and western parts of the country. Nevertheless, the explanatory power and consistency of the five groupings are still high, indicating that the development of the smart health and aging care industry is affected by a combination of multiple factors, showing significant regional heterogeneity and reflecting the uneven development of the digital economy-driven smart health and aging care industry in China. As shown in Figure 3.

Further observation reveals significant regional variations in the explanatory power of high-quality development pathways. This phenomenon primarily stems from China’s uneven regional socioeconomic development levels and the gradient disparities in digital economic advancement. For the underdeveloped regions in central and western China, the digital economy’s explanatory power for the high-quality development of the smart health and aging care industry is relatively limited. The underlying reasons can be summarized across multiple dimensions: First, lagging digital infrastructure development and weak technological application capabilities. Underdeveloped regions exhibit notable shortcomings in network coverage, computing power support, and smart terminal penetration. This hinders the widespread integration of digital technologies into aging care scenarios, thereby limiting the industrial driving effect the digital economy should inherently possess. Second, slow digital organizational transformation and insufficient human capital reserves. Local older adults care enterprises and service providers predominantly adhere to traditional operational models, failing to effectively advance the digital restructuring of organizational structures and business processes. Simultaneously, there is a severe shortage of hybrid talent proficient in both older adults care operations and digital skills, restricting the digital economy’s seamless assimilation and full realization of its benefits in the industry. Third, the policy support system is incomplete, and incentive mechanisms are lacking. Local governments have yet to provide sufficient policy support for the digital economy’s empowerment of the aging care industry. The absence of targeted fiscal subsidies, tax incentives, and additional support hinders effective social capital engagement, restricting the digital economy’s impact on industrial advancement. Fourth, digital financial services suffer from insufficient penetration and inadequate inclusiveness. In underdeveloped regions, digital financial products are limited in variety and service coverage, leaving some older adults groups facing “service blind spots.” Simultaneously, an underdeveloped social credit system constrains innovation and application of digital finance within the older adults care sector, weakening the digital economy’s role as a key driver.

#### 4.2.3 Inter-group results

**Figure 4:**
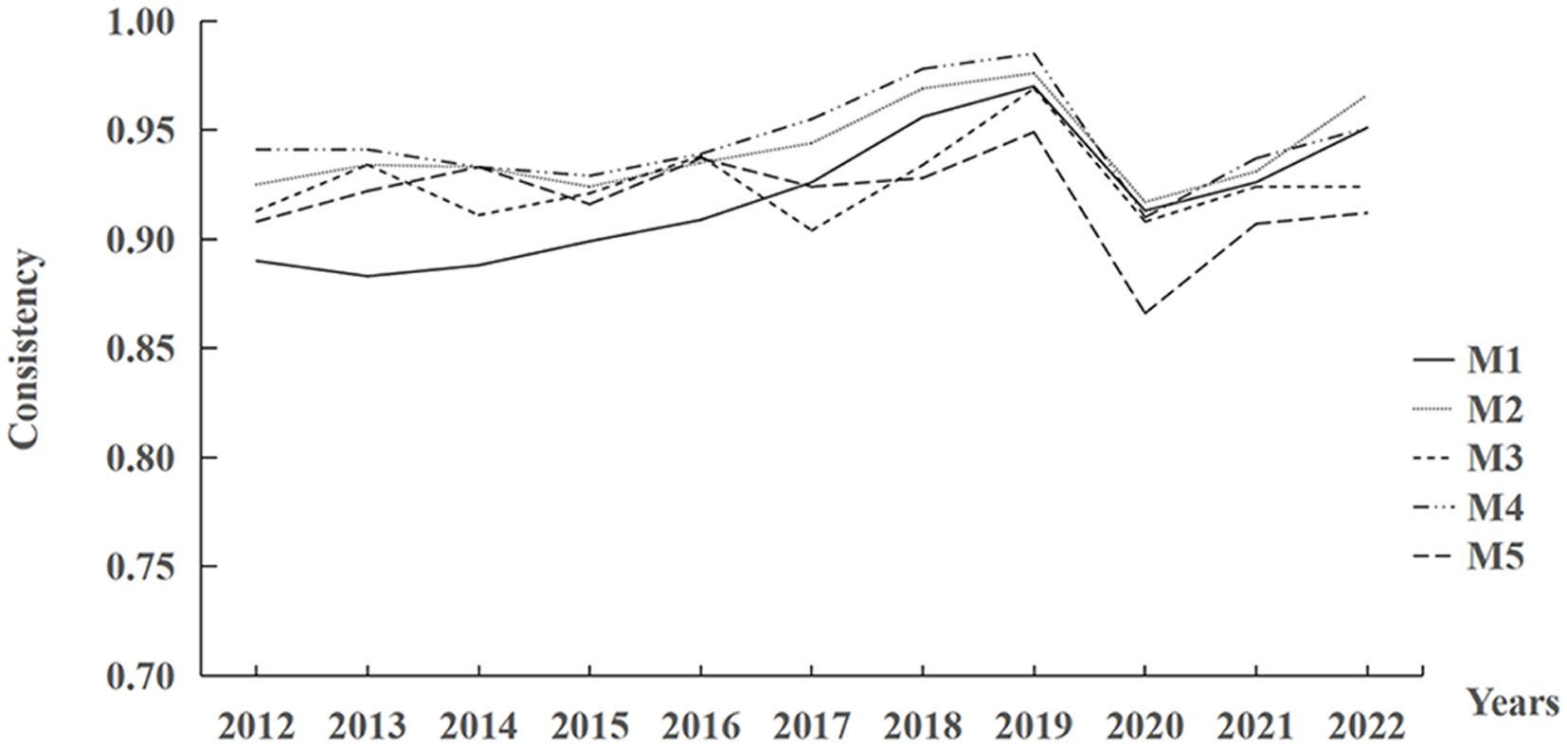
Intergroup consistency levels for configurations M1–M5.

According to Table 4, the data of each grouping path of the digital economy-driven high-quality development of the smart health and aging care industry show that the inter-group consistency coefficients are higher than the 0.75 standard; and the inter-group consistency adjustment distance is less than the threshold of 0.2, indicating that the grouping paths have temporal stability. Analysis of the longitudinal time-series data from 2012 to 2022 reveals that the consistency level of all groupings fluctuates at a high level of 0.85–1.00 and shows a slow upward trend between 2012 and 2019, but collectively shows a decline in 2020. The reason for this is likely to have been influenced by the new Crown Pneumonia epidemic in 2020, resulting in a proportional reduction in the factors’ explanatory efficacy. In a given period, the construction of digital infrastructure was hindered, the application of digital technology was limited, the digital financial environment was volatile, the financing of the pension industry was difficult, the service capacity declined, and the investment of government resources was fragmented. However, because the intergroup consistency adjustment distance is less than 0.2, it does not affect the overall explanatory power. Therefore, the conclusions of this study are reliable and applicable under normal conditions, and the development of the digital economy-driven smart health and aging care industry still has important reference value and generalization. As shown in Figure 4.

### 4.3 Results of multi-temporal comparative analysis

To delve into the intricate progression and key features of the flourishing smart health and longevity sector, powered by the digital economy, this research employs a time-sensitive analytical approach, drawing inspiration from the methodologies of past studies (61). This study divides the timeline into two phases 2012–2016 and 2017-2022—based on China’s five-year planning cycles and the evolution of smart older adults care policies. This division is primarily based on the fact that China’s smart aging care industry began in 2012. It wasn’t until 2017 that three national ministries jointly issued the “Smart Health and Aging Care Industry Development Action Plan (2017–2020),” marking the introduction of the first national-level smart aging care industry plan. This signaled a shift in the industry’s development from a phase of macro-level advocacy and scattered pilot projects to a nationwide promotion phase, forming a policy milestone for dividing the stages. Additionally, examining the dynamic evolution of core variables reveals that during 2012–2016, the primary drivers were the construction of digital infrastructure and preliminary exploratory government policies. From 2017 to 2022, the focus shifted to the release of accumulated digital infrastructure and technological application capabilities. These synergized with organizational innovation, human capital, targeted government policies, and digital finance, collectively propelling the industry from scaled expansion toward a high-quality development paradigm. This division effectively captures the dynamic evolution of causal combinations driving the high-quality development of the smart health and aging care industry, ensuring the logical consistency and robustness of subsequent multi-period QCA comparative analysis. A standardized analysis process was used for each time period: first, the calibration standard was unified; second, the aggregated fsQCA method was used. The results of the necessity condition test show that the aggregated consistency coefficients of the six condition variables in the two time periods do not reach the critical threshold of 0.9, and none of them is a necessary condition to drive the high-quality development of the industry. Subsequently, frequency thresholds for both time periods were set to 1, while original consistency and PRI thresholds were established at 0.80 and 0.70, respectively, and the final enhanced intermediate and parsimonious solution grouping results for the two time periods were obtained. Detailed outcomes are presented in Table 5.

**Table 5:** Multi-temporal analysis of high-quality development groupings in the smart health and aging care industry.

Conditional variable	First period: 2012–2016				Second period: 2017–2022		
	Digital technology–driven	Digital technology–digital environment driven		Digital environment leading	Digital technology–driven	Digital technology–digital environment driven	
	M1	M2	M3	M4	M5	M6	M7
Digital infrastructure	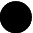	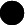			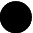	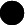	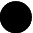
Digital technology application	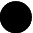	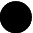	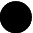		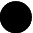	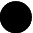	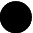
Digital organizational innovation	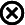			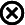	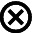	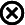	
Digital human capital							
Government digital policy	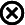		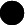	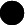	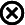	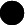	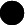
Digital inclusive finance		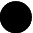	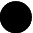	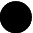	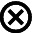	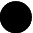	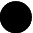
Consistency	0.896	0.925	0.928	0.884	0.963	0.983	0.978
PRI	0.857	0.902	0.906	0.818	0.676	0.833	0.885
Degree of coverage	0.631	0.777	0.771	0.378	0.414	0.397	0.505
Unique coverage	0.010	0.029	0.010	0.052	0.052	0.016	0.505
Inter-group consistency adjustment distance	0.841				0.963		
Intra-group consistency adjustment distance	0.874				0.831		
Overall PRI	0.883				0.609		
Overall consistency	0.854				0.823		
Overall coverage	0.532				0.278		

Large 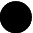 and 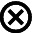 indicate the presence or absence of core conditions, small 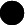 and 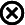 indicates the presence or absence of an auxiliary condition; a blank space indicates that presence or absence is also possible.

Table 5 reveals four classification methods are identified in the first time period and three grouping paths are identified in the second time period. The consistency coefficients of all individual grouping patterns and the overall consistency of the two time periods exceed the threshold judgment criterion of 0.75, and the overall coverage of the two time periods are 0.883 and 0.609, which are able to explain 88.3 and 60.9% of the observed cases, respectively. The multi-temporal evolutionary trajectory patterns were categorized into transitive, buffer-dominant, dominant, and hybrid trajectories (62). According to the evolution pattern of the digital economy-driven high-quality development of the smart health and aging care industry in the two time periods, it can be observed that the digital technology-driven and “digital technology-digital environment” dual-core-promoted grouping patterns have always appeared stably in the two time periods, which implies that the two grouping patterns have an important influence on the high-quality development of the smart health and aging care industry, and their evolution trajectories are the “dominant” trajectories. This means that both models have an important impact on the high-quality development of the smart health and aging care industry, and the trajectory of change is the “dominant trajectory.” On the other hand, the digital environment-led group appeared in the first time period, and also appeared in the second time period, which means that the trajectory of this group has turned, so it is a “turning trajectory.” In addition, multi-temporal comparative analysis can also be used to explore the evolutionary trajectory of individual variables. Based on the cross-temporal dynamic analysis of the six antecedent variables of the digital economy, it is found that the government’s digital policy continues to play a key driving role in promoting the high-quality development of the smart health and aging care industry in the grouping path.

### 4.4 Robustness test

To ensure the robustness of the panel QCA analysis results, this study employed multiple verification methods (63). First, the PRI threshold was adjusted by raising the consistency threshold from 0.70 to 0.75. The revised QCA analysis yielded an overall consistency of 0.893 and a coverage of 0.814. The core condition composition and high-configuration paths remained substantially unchanged after the PRI adjustment. Second, calibration anchor points were adjusted by modifying the quantile values for full membership, cross-membership, and full non-membership from (95, 50, 5%) to (85, 50, 15%). The overall consistency reached 0.881 with a coverage of 0.797. The reconfigured results remained largely consistent with the original configuration. Third, by dividing the research cycle into three phases for dynamic analysis and adjustment: 2012–2015, 2016–2019, and 2020–2022. The overall consistency values for each phase were 0.837, 0.815, and 0.823, respectively, with coverage rates of 0.373, 0.421, and 0.245. Despite the finer temporal segmentation, the core conditions and key pathways driving the high-configuration trajectory remained largely consistent across different time periods, with its evolutionary trajectory remaining clearly discernible. These systematic tests collectively demonstrate that the conclusions drawn in this study exhibit strong robustness to parameter settings and period divisions, yielding reliable results. Detailed outcomes are depicted in Table 6.

**Table 6:** Robustness test results.

Conditional variable	Digital technology driven	Digital technology–digital environment facilitated			Digital environment leading
	M1	M2	M3	M4	M5
Adjustment of the consistency threshold PRI = 0.8	=	=	≈	=	=
Change of calibration anchors (85 per cent, 50 per cent, 15 per cent)	=	=	=	≈	=
Adjustment time window (2012–2015, 2016–2019, 2020–2022)	≈	=	=	=	≈

= indicates that there is no substantial change in the results; ≈ indicates that the results are close in value and the change is small.

## 5 Discussion

### 5.1 Key findings

This study employs the TOE theoretical framework and dynamic QCA methodology to examine data of 30 Chinese provincial panels over the period 2012 to 2022. It examines the diverse pathways and temporal evolution patterns through which the digital economy drives high-quality development in the smart health and aging care industry, exploring these dynamics across three dimensions: technology, organization, and environment. The findings not only reveal the complex influence mechanisms of different configuration pathways on the high-quality development of the smart health and aging care industry but also identify the evolutionary trajectories of core driving factors across distinct developmental stages.

From a prerequisite analysis perspective, no single factor constitutes a necessary condition for the high-quality development of the smart health and aging care industry. The qualitative leap in this sector fundamentally stems from the synergistic interactions and systemic coupling among multiple elements, rather than being dominated by any single independent variable. This finding resonates with existing research. For instance, Liu et al., noted that in complex socioeconomic systems, aging care industry transformation and development are often driven by multi-level, multi-dimensional factors. Relying solely on technological investment or policy support is insufficient to achieve systemic quality breakthroughs (19). Therefore, examining the interactive relationships and overall configuration among different factors from a configuration perspective offers a more realistic and comprehensive theoretical explanation for understanding the high-quality development pathways of the smart health and aging care industry (40, 41).

Within the configuration pathway, the digitally technology-driven model (M1) highlights the synergistic effects of digital technology applications and digital infrastructure in driving the high-quality development of the smart health and aging care industry. This conclusion aligns with the findings of Ding et al., indicating that the competitiveness of smart aging care industry development depends on the depth of embedding and breadth of coverage of digital technologies and their infrastructure within the industrial system (16). Unlike the research by Liu and Xu, they study by confirms the crucial role of industrial chain innovation in the relationship between digital technology application and the development of the smart health and aging care industry (19). Digital technologies drive systematic innovation and development by integrating the upstream, midstream, and downstream segments of the health and aging care industrial chain (21, 34). This study further expands this perspective by focusing on how digital technology applications synergize with other key elements to collectively influence the high-quality development of the smart health and aging care industry. Concurrently, digital infrastructure serves as the foundational support, utilizing advanced information technologies including 5G and IoT to ensure the inter connectivity of industrial data and the efficient implementation of services (36, 38). Through these mechanisms, digital technology applications and digital infrastructure jointly form an integrated driving force propelling the sustainable development of the smart health and aging care industry.

The dual-core “digital technology-digital environment” driving model (M2, M3, M4) emphasizes the synergistic effects of digital technology applications, digital infrastructure, government digital policies, and digital inclusive finance. Existing research confirms that these elements significantly influence the development of the smart health and aging care industry (40, 41). For instance, Liu and Xu notes that the deep integration of smart terminals with health cloud platforms makes digital technology applications a key driver for the smart aging care industry (19). Hou and Li demonstrate that digital infrastructure—represented by high-speed networks and the Internet of Things—not only ensures the continuity and reliability of smart older adults care development but also builds foundational support for industrial innovation (20). Gomber et al., determined that areas with greater concentrations of digital inclusive finance are more likely to foster a virtuous industrial ecosystem through policy guidance, thereby validating the synergistic effects between digital policies and financial instruments (50). This study further reveals that synergies between digital technologies and digital environments are particularly pronounced in contexts where digital organizational capabilities are weak but policy direction is clear. Digital technologies provide the “hard power” for industrial upgrading, while policy and financial environments constitute the “soft power” for sustainable development. Consequently, even with relatively scarce organizational resources, regions can achieve leapfrog development in the aging care industry by building systematic digital ecosystems.

The digital environment-driven model (M5) emphasizes the synergistic effects of government digital policies and digital inclusive finance. Against the backdrop of weak industrial foundations and immature technology applications, governments guide resources toward the smart health and aging care sector through forward-looking policy planning (45, 47). Concurrently, digital inclusive finance effectively enhances the sector’s inclusiveness and sustainability by lowering service access barriers. This synergistic mechanism between digital policy and finance is supported by existing research. For instance, Yang note that robust data security and privacy protection regulations not only help regulate market order but also activate the industry’s endogenous momentum by enhancing user trust (52). In this process, digital inclusive finance provides indispensable financing support for small and micro enterprises to participate in market competition, thereby strengthening their market survival and development capabilities (50, 51). Thus, within this pathway, the synergy between government digital policies and digital inclusive finance not only fortifies the risk resilience of industrial systems but also injects sustained momentum toward achieving more inclusive long-term growth.

Consistent with existing research findings, this study further confirms the pivotal role of digital technologies in driving the development of the smart health and aging care industry. It highlights that, in the long term, the continuous iteration and integrated application of technologies are crucial for addressing the challenges of healthy aging and enhancing the quality of aging care services (16, 19, 21, 36). Moreover, this research underscores the twofold effect of environmental ambiguity on the progression of the intelligent health aging sector. On the one hand, as a vital driver of industrial growth, proactive government digital policies can accelerate the implementation and promotion of smart health industry solutions by improving digital infrastructure and fostering a favorable institutional environment (45, 46). On the other hand, the deepening development of digital inclusive finance can effectively alleviate financing constraints during the industry’s early stages and broaden service coverage (50). However, in environments with high uncertainty, fluctuations in digital inclusive finance may also “induce” some enterprises to over-rely on short-term funding support, thereby weakening the intrinsic momentum for the development of the smart health and aging care industry (52).

Beyond the aforementioned analysis, this study further reveals through multi-period comparative analysis that the digitally technology-driven configuration and the dual-core “digital technology-digital environment” configuration persist stably across both periods, exhibiting strong path dependency. Their evolutionary patterns align with the “dominant trajectory.” This indicates that sustained technological accumulation and digital ecosystem development provide fundamental support for the high-quality advancement of the smart health and aging care industry. In contrast, the digitally environment-led configuration, emerging in the first period, underwent structural adjustments and path renewal in the second period. Its transformation pattern exhibits a typical “turning-point trajectory,” reflecting that development paths driven by external policy environments require dynamic adaptation to changing macro-conditions. Furthermore, this research uncovers substantial spatial variation in the influence of digital economy catalysts. In eastern coastal regions, digital inclusive finance and digital technology applications exert particularly pronounced impacts on the smart health and aging care industry. This is closely linked to their higher economic levels, mature digital infrastructure, and talent aggregation effects. In contrast, central and western regions, constrained by resource endowments and institutional environments, exhibit relatively slower penetration of digital technologies and services. They rely more heavily on policy guidance and foundational environment construction to achieve localized breakthroughs. This finding aligns with regional innovation system theory, which posits that disparities in resource distribution and developmental stages across regions determine the diversity and specificity of their industrial development pathways (19, 23).

### 5.2 Theoretical and practical significance

The theoretical significance of this study lies primarily in three aspects: First, it deepens and expands the theoretical implications of the TOE framework within the context of the smart health and aging care industry. While the traditional TOE framework systematically identifies the influence of technology, organization, and environment, it fails to clarify their synergistic logic within the dynamic evolution of specific industries. This study reveals that no single TOE element constitutes a necessary condition for high-quality industrial development. This finding strongly confirms that in the digital economy era, the smart health and aging care industry follows a complex causal logic characterized by “multiple concurrent paths” and “convergence toward a common goal.” This conclusion breaks the traditional analytical mindset of seeking a single core driver, emphasizing the nonlinear, synergistic interplay among technological application, organizational transformation, and institutional environment. It thereby enriches the TOE framework’s capacity to explain the dynamic mechanisms underlying the formation and development of emerging cross-sector industries.

Second, this study provides key empirical evidence from the smart health and aging care sector for understanding the relationship between digital governance ecosystems and high-quality industrial development. While existing research often highlights the importance of digital technology itself, it lacks detailed exploration of the macro-governance ecosystem models that support its implementation. The three high-quality pathways identified in this study: digital technology-driven, dual-core “digital technology-digital environment” driven, and digital environment-led along with two low-quality traps: the “digital technology-digital environment” constrained and the multidimensional “digital technology-digital organization-digital environment” constrained essentially reflect the differing efficacies of digital governance ecosystems. This demonstrates that a high-quality industrial digital ecosystem can be either strongly technology-driven or carefully environment-nurtured, providing a precise theoretical framework for constructing multi-stakeholder, multi-level collaborative governance models in practice.

Third, this study responds to management scholarship’s call for incorporating the “time” dimension into configurational analysis and demonstrates its methodological value. Static QCA analysis struggles to capture the dynamic evolution of driving pathways, risking theoretical stagnation. By adopting dynamic QCA, this study not only identifies driving pathways but also traces their evolutionary trajectories over time (e.g., “dominant trajectories” and “transitional trajectories”) and their heterogeneous spatial distributions across cross-sections. This methodological advancement enables research findings to reveal more complex dynamic coupling relationships between digital economic development and industrial evolution, significantly enhancing theoretical saturation and the robustness of conclusions. It provides a valuable template for subsequent studies engaged in diachronic, process-oriented configuration research. Second, this study provides crucial empirical evidence from the smart health and aging care sector for understanding the relationship between digital governance ecosystems and high-quality industrial development. Existing research often emphasizes the importance of digital technologies themselves while lacking detailed exploration of the macro-level governance ecosystem models that support their implementation. The three high-quality pathways identified in this study: digital technology-driven, dual-core “digital technology-digital environment” propulsion, and digital environment-led, alongside two low-quality traps: constrained “digital technology-digital environment” and multidimensionally restricted “digital technology-digital organization-digital environment,” essentially reflect varying levels of digital governance ecosystem efficacy. This demonstrates that a high-quality industrial digital ecosystem can be either strongly technology-driven or carefully environment-nurtured, thereby providing a precise theoretical framework for constructing multi-stakeholder, multi-level collaborative governance models in practice.

Additionally, this study’s results provide three practical applications. First, they provide a theoretical basis and practical framework for governments at all levels to formulate differentiated and targeted industrial promotion policies. This research reveals that digital technology and digital environment are core drivers of high-quality industrial development, yet their pathways exhibit significant regional heterogeneity. Consequently, policy creation must eschew a universal solution method. For eastern coastal provinces with robust digital infrastructure and dense technical talent pools, the focus should be on deepening digital technology applications and refining digital inclusive financial systems to foster a technology-driven development model. For central and western provinces, priority should be given to optimizing the digital policy environment and strengthening digital infrastructure to cultivate a “digital ecosystem” conducive to industrial incubation and growth, pursuing an environment-nurturing pathway.

Second, it identifies key strategic resource allocation directions and potential risks for various industry participants, including aging care service providers and technology companies. The study reveals that no single factor can independently drive development; enterprises must seek synergistic alignment between technology and the environment based on their regional ecosystem patterns. For instance, in the dual-core “digital technology-digital environment” driven pathway, enterprises should focus on deeply integrating technological R&D with local policy orientations and financial support. Simultaneously, the low-quality development pathways identified in the study serve as a warning to market entities. They suggest that enterprises should avoid blind investment under scenarios of dual constraints in “digital technology-digital environment” or lagging organizational innovation. Instead, they should proactively seek integration into regional collaborative ecosystems to circumvent multidimensional development traps.

Third, it provides a temporal perspective and early warning indicators for evaluating and adjusting industrial development strategies within dynamic processes. The “dominant trajectories” and “transition trajectories” identified in this study indicate that the driving models of the smart health and aging care industry evolve over time. Notably, the widespread decline in configuration consistency observed in 2020 suggests that major external shocks, such as public health events can inflict systemic impacts on the industry’s digital ecosystem. This necessitates that managers establish dynamic monitoring mechanisms to closely track the stability of key core conditions. When signs of diminishing effectiveness emerge in the dominant pathway’s driving effects, decision-makers should be able to gain timely insights. By referencing the experience of the “transition trajectory,” they can guide the industry to shift from a single technological or environmental pathway toward a more robust “dual-core propulsion” model, thereby enhancing the resilience and sustainability of industrial development.

### 5.3 Limitations and future directions

This research has specific limitations. First, at the theoretical framework level, it analyzes the driving forces behind the smart health and aging care industry primarily from a supply-side perspective (technology, organization, environment) via the TOE framework. While the study validates the pivotal impact of elements like digital technology and its environment, the framework fails to systematically incorporate demand-side factors, such as the digital literacy of older adults and their heterogeneous service needs (64). This limitation primarily stems from the challenges in obtaining micro-level individual data in current research. Consequently, the study’s conclusions may emphasize depicting the “supply potential” of industrial development while failing to fully reveal the obstacles technology may encounter in actual implementation due to the “digital divide.” Second, regarding variable measurement, constrained by the availability and uniformity of macro-level data, proxy indicators for certain variables may not accurately or comprehensively capture their theoretical essence. For instance, the measurement of “digital organizational innovation” and “digital human capital” requires further refinement, which to some extent limits the possibility of more nuanced characterization of the digital economy’s impact mechanisms. Third, at the research data level, while the provincial panel data effectively captures macro trends, its granularity remains insufficient. It fails to reveal the heterogeneity and dynamic micro-level mechanisms within provinces, particularly between cities or at the community level, limiting the interpretive power and practical guidance of the findings at the micro level.

Given these limitations, future research may explore breakthroughs in multiple directions. First, regarding theoretical frameworks, subsequent studies could integrate established theoretical perspectives (such as dynamic capability theory and innovation ecosystem theory) or incorporate demand-side variables like digital literacy and usage willingness among older adult users. This would establish a comprehensive analytical framework balancing supply and demand, enabling more precise prediction and evaluation of the social benefits within the smart health and aging care industry. This would address the theoretical boundaries of the TOE framework, revealing more comprehensively the diverse pathways and potential “blind spot” factors driving high-quality development in the smart health and aging care industry from both supply and demand dimensions. Second, regarding variable measurement and data, future research should focus on developing more precise, multi-level indicator systems and advancing research data to the micro level. On the one hand, data collection should extend to the municipal level and even down to the enterprise/institutional level, utilizing more advanced econometric methods for validation. On the other hand, it is strongly recommended to incorporate qualitative research methods (such as multi-case comparisons, in-depth interviews, and participant observation) to gather first-hand textual and narrative data through in-depth investigations of policymakers, community managers, aging care service providers, and senior users. This strategy of combining “macro-level quantitative characterization” with “micro-level qualitative analysis” will help truly unravel the “black box” of the intrinsic role of the digital economy in driving the development of the smart health and aging care industry. It will enhance our insight into its intricate cause-and-effect processes and lay a robust conceptual groundwork for developing more nuanced and targeted implementation strategies.

## 6 Conclusion

### 6.1 Research findings

This study employs the TOE framework and dynamic QCA methodology, employing panel data from 30 Chinese provinces for the period 2012–2022. It systematically examines the multiple pathways and dynamic evolution through which the digital economy drives high-quality development in the smart health and aging care industry from a configuration perspective. Findings reveal: First, necessity analysis indicates that none of the six digital economy variables covered by the TOE framework passed the necessity test. This suggests that both high-quality and low-quality development in the smart health and aging care industry stem not from a single factor but depend on the synergistic interaction of multiple elements. Second, five high-quality development pathways were identified, categorized into three models: digital technology-driven, dual-core “digital technology-digital environment” propulsion, and digital environment-led. Low-quality development pathways manifested as “digital technology-digital environment” constraint-type and multidimensional “digital technology-digital organization-digital environment” limitation-type. Third, temporal evolution revealed a significant decline in the consistency of high-quality configurations in 2020. spatially, provinces exhibited pronounced regional heterogeneity in configuration coverage. Finally, multi-period comparative analysis further revealed multiple evolutionary trajectories: the digital technology-driven and dual-core “digital technology-digital environment” models constitute the “dominant trajectory,” while the digital environment-led model represents a “transitional trajectory.” In summary, the digital economy’s driving force for high-quality development in the smart health and aging care industry follows a complex causal logic of “multiple concurrent paths” converging toward a common destination. This finding not only deepens theoretical understanding of industrial digitalization but also provides empirical evidence for implementing differentiated, dynamic industrial policies in the smart health and aging care sector.

### 6.2 Policy recommendations

This study proposes the following three differentiated policy recommendations: First, eastern regions should focus on technological leadership and environmental optimization to establish innovation hubs for the smart health and aging care industry. Eastern regions possess significant first-mover advantages in digital technology application and digital inclusive finance. Policies should drive two key initiatives: First, encourage the deep integration and model innovation of cutting-edge technologies like artificial intelligence, the Internet of Things, and big data in smart health and aging care scenarios such as home-based care, chronic disease management, and rehabilitation assistance. Support the development of a group of demonstrative, comprehensive benchmark industrial projects. Second, guide financial institutions and technology companies to collaboratively develop scenario-based, customized digital financial products such as older adults care insurance, smart wellness trusts, and rehabilitation wealth management. This will build an inclusive financial service ecosystem covering the entire life cycle, achieving a leap in capability from technology empowerment to model innovation, and from financial services to ecosystem construction.

Second, the central region should strengthen organizational transformation and efficiency enhancement to stimulate the intrinsic momentum of the smart health and aging care industry. While the central region has established a certain foundation in digital infrastructure, its overall development efficiency is constrained by insufficient digital organizational innovation capabilities. Policies should focus on bridging the “last mile” of digital transformation: by establishing dedicated subsidies for the digital transformation of smart health and aging care institutions, implementing star-rating evaluations and model selection initiatives, and encouraging various older adults care service providers to undertake organizational process reengineering and digital management upgrades. Support should be provided for community-based older adults care service centers and private institutions to adopt intelligent management platforms, deliver integrated online-offline health and older adults care services, and enhance data-driven operational management and resource allocation capabilities. This will effectively translate infrastructure advantages into service efficiency and organizational competitiveness.

Third, western regions should focus on addressing infrastructure gaps and cultivating talent to solidify the foundation for the smart health and aging care industry. Western provinces commonly face dual bottlenecks of inadequate digital infrastructure coverage and shortages in digital human capital. Policies should prioritize “strengthening foundations and cultivating talent”: On the one hand, implement a Western Digital Infrastructure Initiative to extend 5G networks and gigabit fiber-optic coverage to rural and remote areas. Leverage regional hub nodes to deploy centralized, green computing resources, providing stable physical support for smart health and aging care applications. On the other hand, launch the Silver-Haired Digital Talent Revitalization Program. Through targeted recruitment, tuition compensation, on-the-job training, and continuing education, systematically cultivate versatile professionals with both healthcare expertise and digital application skills, providing robust talent support for the industry’s sustainable development.

## Data Availability

The original contributions presented in the study are included in the article/Supplementary material, further inquiries can be directed to the corresponding author.

## References

[1] National bureau of statistics of the People’s Republic of China. *Seventh national population census bulletin (no. 5)-population age composition*. (2021). Available online at: nhttps://www.gov.cn/guoqing/2021-05/13/content_5606149.htm (Accessed March 22, 2025).

[2] Bao J Zhou L Liu G Tang J Lu X Cheng C . Current state of care for the elderly in China in the context of an aging population. Biosci Trends. (2022) 16:107–18. doi: 10.5582/bst.2022.01068, 35431289

[3] Peng R Huang J Deng X Wang Y. Spatial differentiation and driving factors of the high-quality development of undertakings for the aged of China. Int J Equity Health. (2023) 22:104. doi: 10.1186/s12939-023-01921-7, 37237399 PMC10214618

[4] Wang M Qi X Li Z Li J Dong S. Evaluation of the suitability of elderly care in prefecture-level cities in China based on GIS. Heliyon. (2023) 9:539. doi: 10.1016/j.heliyon.2023.e16539, 37303514 PMC10250761

[5] Zhu H Walker A. The gap in social care provision for older people in China. Asian Soc Work Policy Rev. (2018) 12:17–28. doi: 10.1111/aswp.12134

[6] Yi J Lu D Deng Y. The future of social elderly care in China: from the perspective of service-oriented government. J Serv Sci Manag. (2016) 9:211. doi: 10.4236/jssm.2016.93025

[7] State Council of the People’s Republic of China. *Outline of the “healthy China 2030” program*. (2016). Available online at: https://www.gov.cn/zhengce/202203/content_3635233.htm (Accessed March 22, 2025).

[8] Kolesnikov AV Zernova LE Degtyareva VV Panko IV Sigidov YI. Global trends of the digital economy development. Opción Rev Cienc Hum Soc. (2020) 1:523–40. Available online at: https://dialnet.unirioja.es/servlet/articulo?codigo=7827040

[9] United Nations. Digital economy report 2024: Shaping an environmentally sustainable and inclusive digital future. New York: United Nations (2024).

[10] Sharma A Harrington RA McClellan MB Turakhia MP Eapen ZJ Steinhubl S . Using digital health technology to better generate evidence and deliver evidence-based care. J Am Coll Cardiol. (2018) 71:2680–90. doi: 10.1016/j.jacc.2018.03.523, 29880129

[11] Ministry of Industry and Information Technology of the People’s Republic of China, Ministry of Civil Affairs, National Health and Health Commission. *Circular on the issuance of the action plan for the development of the smart healthy aging industry (2021–2025)*. (2021). Available online at: https://www.gov.cn/zhengce/zhengceku/2021-10/23/content_5644434.htm (Accessed March 21, 2025).

[12] State council of the People’s Republic of China. *Circular on the issuance of the 14th five-year plan for the development of the digital economy*. (2022). Available online at: https://www.gov.cn/xinwen/2022-01/12/content_5667840.htm (Accessed March 21, 2025).

[13] General office of the state council of the People’s Republic of China. *Opinions on developing the silver-hair economy and enhancing the well-being of the elderly*. (2024). Available online at: https://www.gov.cn/zhengce/content/202401/content_6926087.htm (Accessed March 21, 2025).

[14] Xia L Baghaie S Sajadi SM. The digital economy: challenges and opportunities in the new era of technology and electronic communications. Ain Shams Eng J. (2024) 15:102411. doi: 10.1016/j.asej.2023.102411

[15] Wang J. Research on empowering high-quality development of elderly care service industry with digital economy. Inf Syst Econ. (2024) 5:92–7. doi: 10.23977/infse.2024.050213

[16] Ding Z Qu X Li C. Digital economy and high-quality development of the healthcare industry. Front Public Health. (2024) 12:1331565. doi: 10.3389/fpubh.2024.1331565, 38282760 PMC10820708

[17] Guo B Wang Y Zhang H Liang C Feng Y Hu F. Impact of the digital economy on high-quality urban economic development: evidence from Chinese cities. Econ Model. (2023) 120:106194. doi: 10.1016/j.econmod.2023.106194

[18] Xia J Chen T. Research on how the digital economy contributes to achieving a high-level and balanced development of the elderly care service industry. China Financ Econ Rev. (2024) 13:113–29. doi: 10.1515/cfer-2024-0018

[19] Liu X Xu Y. Research on the growth path of digital economy-enabled senior care industry. Popul Econ. (2024) 4:45–58.

[20] Hou Z Li Y. Mechanisms and pathways for promoting the development of the elderly care industry through the digital economy. Int Rev Econ Finance. (2025) 103:104545. doi: 10.1016/j.iref.2025.104545

[21] Oderanti FO Li F Cubric M Shi X. Business models for sustainable commercialisation of digital healthcare (eHealth) innovations for an increasingly ageing population. Technol Forecast Soc Change. (2021) 171:120969. doi: 10.1016/j.techfore.2021.120969

[22] Chen Y Xu Y. The influencing factors and improvement paths of the manufacturing industry innovation system of products for the elderly. Math Probl Eng. (2022) 2022:1174622. doi: 10.1155/2022/1174622

[23] Wei Y Chen Y Wang X. Study on the measurement of high-quality development and regional differences of smart healthy aging industry. Stat Inf Forum. (2024) 39:62–76.

[24] Liu H Wang W Li S. Spatio-temporal evolution and driving factors of the coupling and coordinated development of China’s digital economy and older adult care services. Front Public Health. (2025) 13:1490461. doi: 10.3389/fpubh.2025.1490461, 40078756 PMC11897250

[25] Komathi W Sim CH. Shaping a digital future: examining technology, organization and environment (TOE) framework. J Technol Manag Bus. (2024) 11:80–97. doi: 10.30880/jtmb.2024.11.01.005

[26] Notice of the Ministry of Industry and Information Technology. *The Ministry of Civil Affairs, and the National Health Commission on issuing the “action plan for the development of the smart health and elderly care industry (2021–2025)”*. (2025). Available online at: https://www.gov.cn/zhengce/zhengceku/2021-10/23/content_5644434.htm (Accessed November 17, 2025).

[27] Wang F Wang JD. Investing preventive care and economic development in ageing societies: empirical evidences from OECD countries. Heal Econ Rev. (2021) 11:18. doi: 10.1186/s13561-021-00321-3, 34086126 PMC8176873

[28] Liu S Zhu S Hou Z Li C. Digital village construction, human capital and the development of the rural older adult care service industry. Front Public Health. (2023) 11:1190757. doi: 10.3389/fpubh.2023.1190757, 37546306 PMC10400453

[29] Wang Y Kung L Gupta S Ozdemir S. Leveraging big data analytics to improve quality of care in healthcare organizations: a configurational perspective. Br J Manage. (2019) 30:362–88. doi: 10.1111/1467-8551.12332

[30] Hu Y Wang J Nicholas S Maitland E. The sharing economy in China’s aging industry: applications, challenges, and recommendations. J Med Internet Res. (2021) 23:e27758. doi: 10.2196/27758, 34255691 PMC8293162

[31] Causio FA Hoxhaj I Beccia F Marcantonio MD Strohäker T Cadeddu C . Big data and ICT solutions in the European Union and in China: a comparative analysis of policies in personalized medicine. Digit Health. (2022) 8:129060. doi: 10.1177/20552076221129060, 36329830 PMC9623355

[32] Furnari S Crilly D Misangyi VF Greckhamer T Fiss PC Aguilera RV. Capturing causal complexity: heuristics for confiaurational theorizing. Acad Manag Rev. (2020) 46:778–99. doi: 10.5465/amr.2019.0298

[33] Baker J. The technology–organization–environment framework In: Baker J, editor. Information systems theory: Explaining and predicting our digital society, vol. 1. New York: Springer (2021). 231–45.

[34] Padikkapparambil J Ncube C Singh KK Singh A. Internet of things technologies for elderly health-care applications In: Balas VE Solanki VK Kumar R, editors. Emergence of pharmaceutical industry growth with industrial IoT approach. Cambridge, MA: Academic Press (2020). 217–43.

[35] Liu Y Zhang L Yang Y Zhou L Ren L Wang F . A novel cloud-based framework for the elderly healthcare services using digital twin. IEEE Access. (2019) 7:49088–101. doi: 10.1109/ACCESS.2019.2909828

[36] Zhu Y Yang Q Mao X. Global trends in the study of smart healthcare systems for the elderly: artificial intelligence solutions. Int J Comput Intell Syst. (2023) 16:105. doi: 10.1007/s44196-023-00283-w

[37] Kuo MH Wang SL Chen WT. Using information and mobile technology improved elderly home care services. Health Policy Technol. (2016) 5:131–42. doi: 10.1016/j.hlpt.2016.02.004

[38] He W Liu H Wang M. The spatial impacts of construction of digital village on the development of county-level care industry for older people. China Agric Econ Rev. (2025) 17:795–824. doi: 10.1108/CAER-09-2024-0274

[39] Iljashenko O Bagaeva I Levina A. *Strategy for establishment of personnel KPI at health care organization digital transformation*. IOP conference series: Materials science and engineering. IOP Publishing, p. 12029. (2019).

[40] Zheng S Appolloni A Lin H Ding X. Configuration analysis of the innovation pathway of gerontechnological enterprises under the market-organization-technology perspective. Eur J Innov Manag. (2024) 27:2944–65. doi: 10.1108/EJIM-03-2023-0209

[41] Lu L Liang C Gu D Ma Y Zhao S. What advantages of blockchain affect its adoption in the elderly care industry? A study based on the technology-organisation-environment framework. Technol Soc. (2021) 67:101786. doi: 10.1016/j.techsoc.2021.101786

[42] Feng G Li Q. The study on innovative development of the elderly care industry under the community-based elderly care model based on the servqual model. Open Public Health J. (2025) 18:3. doi: 10.2174/0118749445370791250203060003

[43] Cannavacciuolo L Capaldo G Ponsiglione C. Digital innovation and organizational changes in the healthcare sector: multiple case studies of telemedicine project implementation. Technovation. (2023) 120:102550. doi: 10.1016/j.technovation.2022.102550

[44] Yang J Luo B Zhao C Zhang H. Artificial intelligence healthcare service resources adoption by medical institutions based on TOE framework. Digital Health. (2022) 8:6034. doi: 10.1177/20552076221126034, 36211801 PMC9537501

[45] Zhang X Lu Z Zhu D Zhang Y. How do policies promote the sustainable development of older-adult care industry? A configuration analysis based on policy tools. Front Public Health. (2024) 12:1430679. doi: 10.3389/fpubh.2024.1430679, 39463902 PMC11512454

[46] Feng Z Glinskaya E Chen H Gong S Qiu Y Xu J . Long-term care system for older adults in China: policy landscape, challenges, and future prospects. Lancet. (2022) 396:1362–72. doi: 10.1016/S0140-6736(20)32136-X, 34338215

[47] Xu F Huang Y Wang Q. Aging industries in the regional economy: how to support an aging China? Land. (2022) 11:2096. doi: 10.3390/land11112096

[48] Chen H Zhang Y Wang L. A study on the quality evaluation index system of smart home care for older adults in the community—based on Delphi and AHP. BMC Public Health. (2023) 23:411. doi: 10.1186/s12889-023-15262-1, 36859259 PMC9975439

[49] Frischer R Krejcar O Maresova P Fadeyi O Selamat A Kuca K . Commercial ICT smart solutions for the elderly: state of the art and future challenges in the smart furniture sector. Electronics. (2020) 9:149. doi: 10.3390/electronics9010149

[50] Gomber P Koch JA Siering M. Digital finance and fintech: current research and future research directions. J Bus Econ. (2017) 87:537–80. doi: 10.1007/s11573-017-0852-x

[51] Jena R. Factors impacting senior citizens’ adoption of E-banking post COVID-19 pandemic: an empirical study from India. J Risk Financ Manag. (2023) 16:380. doi: 10.3390/jrfm16090380

[52] Yang D. China’s pension finance under the background of digital transformation research on promoting high-quality economic developmen. J Soc Sci Dev Res. (2024) 2:73–86.

[53] García-Castro R Ariño MA. A general approach to panel data set-theoretic research. J Adv Manag Sci Inf Syst. (2016) 2:63–76. doi: 10.6000/2371-1647.2016.02.06

[54] Hu J Zhang H Irfan M. How does digital infrastructure construction affect low-carbon development? A multidimensional interpretation of evidence from China. J Clean Prod. (2023) 396:136467. doi: 10.1016/j.jclepro.2023.136467

[55] Tsou HT Chen JS. How does digital technology usage benefit firm performance? Digital transformation strategy and organisational innovation as mediators. Technol Anal Strateg Manag. (2023) 35:1114–27. doi: 10.1080/09537325.2021.1991575

[56] Gong S Jiang L Yu Z. Can digital human capital promote farmers’ willingness to engage in green production? Exploring the role of online learning and social networks. Behav Sci (Basel). (2025) 15:227. doi: 10.3390/bs15020227, 40001859 PMC11852135

[57] Gan T Zhang M Zhang Z. The impact of digital government policy on entrepreneurial activity in China. Econ Anal Policy. (2023) 79:479–96. doi: 10.1016/j.eap.2023.06.029

[58] Yu C Jia N Li W Wu R. Digital inclusive finance and rural consumption structure–evidence from Peking University digital inclusive financial index and China household finance survey. China Agric Econ Rev. (2022) 14:165–83. doi: 10.1108/CAER-10-2020-0255

[59] Fiss PC. Building better causal theories: a fuzzy set approach to typologies in organization research. Acad Manag J. (2011) 54:393–420. doi: 10.5465/amj.2011.60263120

[60] Schneider RM Schulze-Bentrop C Paunescu M. Mapping the institutional capital of high-tech firms: a fuzzy-set analysis of capitalist variety and export performance. J Int Bus Stud. (2009) 41:246–66. doi: 10.1057/jibs.2009.36

[61] Wang Y Liu YY. What kind of institutions generate high human capital economic growth effects? A study based on dynamic QCA methodology. J Sci Sci. (2024) 1:289–99. doi: 10.16192/j.cnki.1003-2053.20230413.005

[62] Litrico JB David RJ. The evolution of issue interpretation within organizational fields: actor positions, framing trajectories, and field settlement. Acad Manag J. (2017) 60:986–1015. doi: 10.5465/amj.2013.0156

[63] Meuer J Rupietta C. A review of integrated QCA and statistical analyses. Qual Quant. (2017) 51:2063–83. doi: 10.1007/s11135-016-0397-z

[64] Hargittai E Piper AM Morris MR. From internet access to internet skills: digital inequality among older adults. Univ Access Inf Soc. (2019) 18:881–90. doi: 10.1007/s10209-018-0617-5

