# Supplementary material for: Research on the path of high-quality development of the smart health and aging care industry driven by digital economy— dynamic QCA analysis based on TOE framework
Source: Front Public Health. 2026 Mar 2;14:1661333. doi: 10.3389/fpubh.2026.1661333 (PMC12990852; doi:10.3389/fpubh.2026.1661333)
Supplement: Supplementary file 1 [file Data_Sheet_1.docx]

**TABLE 1** Description of Variable Indicators

| Level 1 indicators | | | Level 2 indicators | Indicator description |
| --- | --- | --- | --- | --- |
| Conditional variable | Digital  technical | Digital infrastructure | Internet penetration rate | Number of Internet users as a proportion of resident population (%) |
|  |  |  | Telephone penetration rate | Total number of telephones (including mobile phones)/total population in administrative area*100 (units) |
|  |  |  | Length of long-haul fibre-optic cable lines | Length of long-haul fibre-optic cable lines (10,000 km) |
|  |  |  | Internet broadband access ports | Number of Internet broadband access ports (10,000) |
|  |  |  | Number of Internet domain names | Number of Internet domain names (10,000) |
|  |  | Digital technology application | Volume of telecommunication services | Total telecommunication services per capita |
|  |  |  | Software business volume | Software business income per capita |
|  | Digital  organizational | Digital organizational innovation | Number of new product development projects | Number of new product development projects per capita in the region in the current year |
|  |  |  | Expenditure on new product development | Per capita expenditure on new product development in the region in the current year |
|  |  |  | Revenue from sales of new products | Revenue from sales of new products per capita in the region in the current year |
|  |  |  | Patents granted for inventions | Effective patents for inventions per 100 persons in the region in the current year |
|  |  | Digital human capital | Digital economy talent | Percentage of employees in computer services and software |
|  |  |  | Digital Research Talent | Total R&D staff for the year |
|  | Digital  environmental | Government digital policy | Digital Government Digital Economy Focus | Frequency of occurrence of keywords of digital economy in the government work report of that year |
|  |  |  | Financial support for science, technology and innovation | R&D expenditure per capita for the year |
|  |  | Digital inclusive finance | Digital Inclusion Financial Digitisation Index | Peking University Digital Finance Inclusion Index |

**TABLE 1** Description of variable indicators (continued)

| Level 1 indicators | | | Level 2 indicators | Indicator description |
| --- | --- | --- | --- | --- |
| Outcome variable | High-quality development of the smart ageingindustry | Innovative development | Intelligent Healthy Aging Industry Innovation Strength | Internal expenditure on R&D/GDP |
|  |  |  | Smart Healthy Aging Industry Innovators | R& D personnel converted to full-time equivalents |
|  |  |  | Patent Scale of Smart Healthy Aging Industry | Total number of patents granted/resident population of the region |
|  |  |  | Patent Quality in the Smart Healthy Aging Industry | Number of patents granted for inventions/total number of patents granted |
|  |  | Co-ordinated development | Difference between urban and rural consumption | Per capita consumption expenditure of urban residents/per capita consumption expenditure of rural residents |
|  |  |  | Urban/rural income differentials | Urban disposable income per capita/rural disposable income per capita |
|  |  |  | General public budget consumption expenditure on old age than | General public budget pension expenditure/population |
|  |  |  | Percentage of fiscal expenditure on old-age pensions | Fiscal expenditure on old-age pensions/fiscal expenditure |
|  |  | Shared  development | Pension insurance coverage | Number of persons enrolled in basic old-age insurance/number of persons liable to be enrolled |
|  |  |  | Pension coverage | Number of socialised pensioners/population aged 55 and over |
|  |  |  | Number of beds per 1,000 elderly population | Number of beds for the elderly/population aged 65 and over (in thousands) |
|  |  |  | Medical insurance participation rate | Number of persons enrolled in basic health insurance/total number of persons |
|  |  |  | Health technicians per 1,000 population | Number of health technicians/population (thousands) |
|  |  |  | Number of community services and facilities per 10,000 population | Number of community services and facilities/population (10,000) |
|  |  |  | Qualified social workers per 1,000 elderly population | Cumulative number of qualified social workers/population aged 65 and over (in thousands) |
|  |  | Sustainable development | Elderly population dependency ratio | Number of persons aged 65 and over/number of persons aged 15-64 in the labour force |
|  |  |  | Labour force literacy | Average years of schooling of the population aged 15 years and over |
|  |  |  | Internet penetration | Internet broadband subscribers/population |
|  |  |  | Primary care load | Number of people aged 65 and over/number of primary care facilities |
|  |  | Efficient development | Concentration of Fixed Asset Investment in Smart Healthy Aging Industry | Investment in fixed assets in industry/investment in fixed assets in the tertiary sector |
|  |  |  | Intelligent Healthy Aging Industry Employee Density | Number of persons employed in industry/population |
|  |  |  | Smart Healthy Aging Industry Capital Productivity | Industrial value added/industrial fixed asset investment |
|  |  |  | Labour productivity in the smart health care industry | Value added of industry/number of people employed in industry |
|  |  |  | GDP Contribution of Smart Healthy Aging Industry | Incremental industrial value added/GDP |

**TABLE 2** Variable calibration results

| Variable classification | Variable name | | Full affiliation | Junction | Totally unaffiliated |
| --- | --- | --- | --- | --- | --- |
| Outcome variable | High-quality Development of Smart Healthy  Aging Industry | | 0.543 | 0.414 | 0.324 |
| Conditional variable | Digital  technical | Digital infrastructure | 0.352 | 0.097 | 0.023 |
|  |  | Digital technology application | 0.157 | 0.030 | 0.003 |
|  | Digital  organizational | Digital organizational innovation | 0.290 | 0.042 | 0.008 |
|  |  | Digital human capital | 0.392 | 0.075 | 0.016 |
|  | Digital  environmental | Government digital policy | 0.339 | 0.133 | 0.026 |
|  |  | Digital inclusive finance | 398.597 | 267.950 | 99.041 |

**TABLE 3** Results of the analysis of the conditions of necessity

| Conditional variable | High Quality Development of Smart Healthy Aging  Industry (Hdi) | | | | Low Quality Development of Smart Healthy Aging  Industry (~Hdi) | | | |
| --- | --- | --- | --- | --- | --- | --- | --- | --- |
|  | Aggregation  Consistency | Summary  Coverage | Intergroup consistency adjustment distance | Intra-group consistency adjustment distance | Aggregation  Consistency | Summary Coverage | Intergroup consistency adjustment distance | Intra-group consistency adjustment distance |
| FsDin | 0.785 | 0.775 | 0.029 | 0.058 | 0.623 | 0.505 | 0.060 | 0.075 |
| ~FsDin | 0.498 | 0.617 | 0.141 | 0.083 | 0.722 | 0.733 | 0.040 | 0.064 |
| FsDta | 0.864 | 0.812 | 0.023 | 0.038 | 0.596 | 0.459 | 0.075 | 0.057 |
| ~FsDta | 0.424 | 0.561 | 0.188 | 0.093 | 0.756 | 0.820 | 0.051 | 0.051 |
| FsDoi | 0.772 | 0.749 | 0.014 | 0.052 | 0.649 | 0.516 | 0.043 | 0.07 |
| ~FsDoi | 0.502 | 0.635 | 0.112 | 0.087 | 0.685 | 0.711 | 0.015 | 0.074 |
| FsDhc | 0.725 | 0.692 | 0.033 | 0.062 | 0.710 | 0.555 | 0.031 | 0.07 |
| ~FsDhc | 0.534 | 0.692 | 0.064 | 0.091 | 0.606 | 0.644 | 0.024 | 0.097 |
| FsGdp | 0.765 | 0.784 | 0.037 | 0.049 | 0.590 | 0.496 | 0.085 | 0.07 |
| ~FsGdp | 0.508 | 0.602 | 0.139 | 0.071 | 0.742 | 0.722 | 0.050 | 0.051 |
| FsDuf | 0.811 | 0.905 | 0.077 | 0.021 | 0.459 | 0.420 | 0.156 | 0.042 |
| ~FsDuf | 0.480 | 0.520 | 0.176 | 0.043 | 0.896 | 0.796 | 0.084 | 0.019 |

**TABLE 4** Results of high/non-high-quality development grouping analysis of the smart health and elderly care industry

| Conditional variable | High Quality Development of Smart Healthy Aging Industry | | | | | Low Quality Development of Smart Healthy Aging Industry | | |
| --- | --- | --- | --- | --- | --- | --- | --- | --- |
|  | Digital technology-driven | Digital Technology - Digital Environment Dual Core Propulsion | | | Digital Environment Leadership | Digital technology-digital environment co-bound type | Digital Technologies - Digital Organisations - Digital Environments Multidimensional Constraints Type | |
|  | M1 | M2 | M3 | M4 | M5 | M6 | M7 | M8 |
| Digital infrastructure | ● | ● |  |  |  |  | ⮿ |  |
| Digital technology application | ● | ● | ● | ● |  | ⮿ | ⮿ | ⮿ |
| Digital organizational innovation | ● | ⮿ | ⮿ |  | ⮿ |  | ⮿ | ⮿ |
| Digital human capital | ⮿ |  | ⮿ |  |  | ● |  |  |
| Government digital policy | ⮿ |  | ● | ● | ● |  |  | ⮿ |
| Digital inclusive finance |  | ● |  | ● | ● | ⮿ | ⮿ | ⮿ |
| consistency | 0.933 | 0.939 | 0.934 | 0.942 | 0.917 | 0.923 | 0.896 | 0.897 |
| PRI | 0.836 | 0.899 | 0.855 | 0.904 | 0.811 | 0.788 | 0.771 | 0.773 |
| Degree of coverage | 0.347 | 0.686 | 0.376 | 0.674 | 0.413 | 0.507 | 0.554 | 0.560 |
| Unique coverage | 0.008 | 0.020 | 0.007 | 0.053 | 0.008 | 0.118 | 0.011 | 0.010 |
| Intergroup consistency  adjustment distance | 0.011 | 0.012 | 0.027 | 0.016 | 0.023 | 0.018 | 0.028 | 0.027 |
| Intra-group consistency  Adjustment distance | 0.029 | 0.029 | 0.029 | 0.036 | 0.030 | 0.019 | 0.026 | 0.026 |
| Overall PRI | 0.819 | | | | | 0.897 | | |
| Overall consistency | 0.888 | | | | | 0.773 | | |
| Overall coverage | 0.810 | | | | | 0.560 | | |

Note: Large ● and ⮿ indicate the presence or absence of core conditions, small ● and ⮿ indicates the presence or absence of an auxiliary condition; a blank space indicates that presence or absence is also possible.

**TABLE 5** Multi-temporal analysis of high-quality development groupings in the smart health and elderly care industry

| Conditional variable | First period: 2012-2016 | | | | Second period: 2017-2022 | | |
| --- | --- | --- | --- | --- | --- | --- | --- |
|  | Digital technology-driven | Digital technology - digital environment driven | | Digital Environment  leading | Digital technology-driven | Digital technology - digital environment driven | |
|  | M1 | M2 | M3 | M4 | M5 | M6 | M7 |
| Digital infrastructure | ⦁ | ⦁ |  |  | ● | ⦁ | ● |
| Digital technology application | ● | ● | ● |  | ● | ● | ● |
| Digital organizational innovation | ⮿ |  |  | ⮿ | ⮿ | ⮿ |  |
| Digital human capital |  |  |  |  |  |  |  |
| Government digital policy | ⮿ |  | ⦁ | ● | ⮿ | ⦁ | ⦁ |
| Digital inclusive finance |  | ● | ● | ● | ⮿ | ● | ⦁ |
| consistency | 0.896 | 0.925 | 0.928 | 0.884 | 0.963 | 0.983 | 0.978 |
| PRI | 0.857 | 0.902 | 0.906 | 0.818 | 0.676 | 0.833 | 0.885 |
| Degree of coverage | 0.631 | 0.777 | 0.771 | 0.378 | 0.414 | 0.397 | 0.505 |
| Unique coverage | 0.010 | 0.029 | 0.010 | 0.052 | 0.052 | 0.016 | 0.505 |
| Intergroup consistency  adjustment distance | 0.841 | | | | 0.963 | | |
| Intra-group consistency  Adjustment distance | 0.874 | | | | 0.831 | | |
| Overall PRI | 0.883 | | | | 0.609 | | |

Note: Large ● and ⮿ indicate the presence or absence of core conditions, small ● and ⮿ indicates the presence or absence of an auxiliary condition; a blank space indicates that presence or absence is also possible.

**TABLE 6** Robustness test results

| Conditional variable | Digital Technology Driven | Digital Technology - Digital Environment  Facilitated | | | Digital Environment leading |
| --- | --- | --- | --- | --- | --- |
|  | M1 | M2 | M3 | M4 | M5 |
| Adjustment of the consistency threshold PRI = 0.8 | = | = | ≈ | = | = |
| Change of calibration anchors (85 per cent, 50 per cent, 15 per cent) | = | = | = | ≈ | = |

Note: = indicates that there is no substantial change in the results; ≈ indicates that the results are close in value and the change is small.
